# Proteasomal Degradation of Zn-Dependent Hdacs: The E3-Ligases Implicated and the Designed Protacs That Enable Degradation

**DOI:** 10.3390/molecules26185606

**Published:** 2021-09-15

**Authors:** Laura Márquez-Cantudo, Ana Ramos, Claire Coderch, Beatriz de Pascual-Teresa

**Affiliations:** Departamento de Química y Bioquímica, Facultad de Farmacia, Universidad San Pablo-CEU, CEU Universities, Urbanización Montepríncipe, Alcorcón, 28925 Madrid, Spain; laura.marquezcantudo@ceu.es (L.M.-C.); aramgon@ceu.es (A.R.)

**Keywords:** PROTACs, HDACs, E3-ligase, drug design, proteasomal degradation

## Abstract

Protein degradation by the Ubiquitin-Proteasome System is one of the main mechanisms of the regulation of cellular proteostasis, and the E3 ligases are the key effectors for the protein recognition and degradation. Many E3 ligases have key roles in cell cycle regulation, acting as checkpoints and checkpoint regulators. One of the many important proteins involved in the regulation of the cell cycle are the members of the Histone Deacetylase (HDAC) family. The importance of zinc dependent HDACs in the regulation of chromatin packing and, therefore, gene expression, has made them targets for the design and synthesis of HDAC inhibitors. However, achieving potency and selectivity has proven to be a challenge due to the homology between the zinc dependent HDACs. PROteolysis TArgeting Chimaera (PROTAC) design has been demonstrated to be a useful strategy to inhibit and selectively degrade protein targets. In this review, we attempt to summarize the E3 ligases that naturally ubiquitinate HDACs, analyze their structure, and list the known ligands that can bind to these E3 ligases and be used for PROTAC design, as well as the already described HDAC-targeted PROTACs.

## 1. Introduction

### 1.1. The Ubiquitin-Proteasome System

Proteins inside the cells are subjected to a well meshed quality control system that is comprised of the Ubiquitin-Proteasome System (UPS), autophagic lysosomes, and the endoplasmic reticulum. Misfolded proteins will reach the endoplasmic reticulum where a refolding process will be attempted by the Heat-Shock Proteins (HSPs). Should this process fail, the misfolded proteins will be transported back to the cytoplasm where they will be finally degraded by either the UPS or lysosomes [1]. Autophagic lysosomes are responsible for the degradation of unwanted protein complexes, insoluble aggregates, and even small organelles [2]; whereas the UPS is responsible not only for marking and degrading misfolded or unwanted proteins, but is also involved in many cellular processes, such as immune function, DNA repair or cell growth [2,3]. The malfunction of any of these systems is linked to the development of different diseases, such as cancer and neurodegenerative diseases, amongst others [4,5].

**Figure 1:**
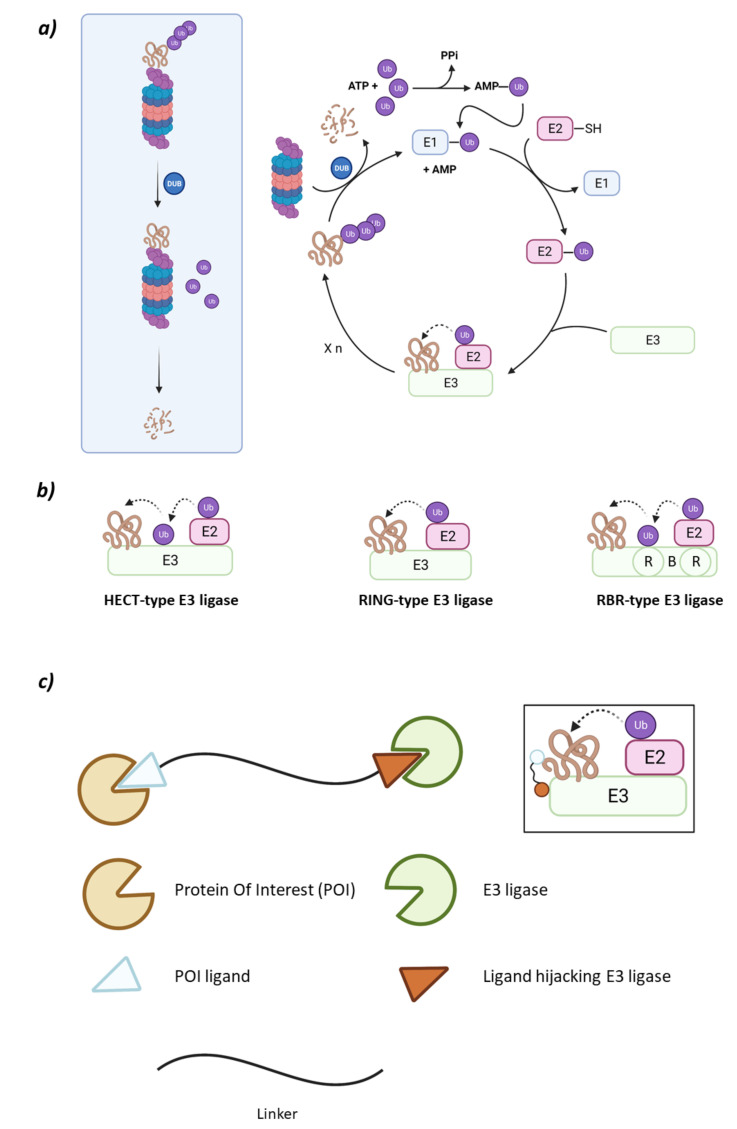
(**a**) Schematic representation of the Ubiquitin-Proteasome System (UPS) for substrate degradation. (**b**) Classification of the E3 ligase families according to their ubiquitination mechanism: HECT-type E3 ligase (indirect), RING-type E3 ligase (direct), and RBR-type E3 ligase (indirect). (**c**) Schematic representation of the overall structure and mechanism of action of PROTACs.

The UPS system is comprised of the 26S proteasome and an ensemble of three enzymes that carry out substrate ubiquitination in cascade: the ubiquitin-activating enzyme (E1), the ubiquitin-conjugating enzyme (E2), and the ubiquitin ligase (E3) [6]. The ubiquitination cascade starts with E1 activating Ubiquitin (Ub) in an ATP-dependent manner so that the activated Ub can be transferred to a cysteine residue in E2. Subsequently, the E3 ligase will transfer Ub either directly or indirectly, depending on the type of E3 ligase, to a lysine residue in the substrate protein through an isopeptide bond formed between epsilon nitrogen in the side chain of lysine and the carboxylic moiety of glycine at the *C*-terminal end of Ub (Figure 1) [7].

Ubiquitination in the substrate protein may happen by either the addition of one Ub molecule on one lysine residue (monoubiquitination) or several lysine residues (multiubiquitination); or the addition of several Ub molecules on one single lysine residue (poliubiquitination) [7]. Poli-Ub chains may be formed by different amounts of Ub molecules connected to each other by different lysine residues giving rise to different types of conformations [8]. Depending on the position of the ubiquitinated lysine residues and the number of Ub molecules attached, the substrate protein will be marked for proteasomal or lysosome degradation, or to fulfill roles in cellular regulation. Of all these possible destinations, when the poli-Ub chain is formed of Lys48, the substrate protein will commonly be targeted by proteasomal degradation, prior to which the 26S proteasome will eliminate the Ub molecules attached [2,7,9].

The type of E3 ligase will determine what protein is going to be ubiquitinated and which is the E2 ligase that is going to take part in the process, as the E3 ligase has specific recognition domains for both proteins [2,6]. There are E2 ligases that are promiscuous and others that have a specific purpose [10]. The E3 ligase family members can be classified according to their tertiary structure in HECT (Homologous to the E6AP Carboxyl Terminus E3 ligases), U-Box, RING (Really Interesting Gene E3 ligases), and RBR (RING-Between-RING E3 ligases) which is a subtype of the RING family [6,11]. However, some authors prefer a different type of classification according to the ubiquitination mechanism that depends on the direct or indirect transfer of the Ub from the E2 to the substrate protein. While the RING E3 ligases favor the direct transfer of Ub from the E2 to the substrate protein working solely as a proximity mediator, the HECT E3 ligases take the Ub from the E2 and bind it to their catalytic cysteine and, in a second step, they transfer the Ub to the substrate. Interestingly, the RBR E3 ligases, despite having two RING domains and being structurally more similar to RING, have a mechanism of action which is more similar to that of the HECT E3 ligases (Figure 1) [9,12]. 

Both the RING and U-box domains are responsible for binding the ubiquitin-carrying E2 ligase (Ub-E2) [13]. Both domains share similar functions as well as tertiary structures, however, unlike the U-box domain [14], RING needs to coordinate zinc ions to maintain the structure. The structure of the RING domain consists of highly conserved zinc-coordinated loops and α-helix that are recognized by the E2 ligase. The Zn^2+^ are coordinated to cysteine residues in the RING finger motif that are recognizable by the following sequence: Cys-X2-Cys-X(9−39)-Cys-X(1−3)-His-X(2−3)-Cys-X2-Cys-X(4−48)-Cys-X2-Cys [15,16]. The RING family can be divided into different subfamilies depending on their associating patterns: Cullin-RING, Monomeric RING, Homodimeric RING, Heterodimeric RING, Monomeric U-box, and Heterodimeric U-box [17]. The Cullin-RING family needs the Cullin protein as a nucleating scaffold for the rest of the ligase system, while Monomeric RING E3 ligases are able to directly bind the E2-ligase and the substrate protein. However, some RING ligases may form homodimers or heterodimers, by either the RING domain or an amino acid sequence outside the above-mentioned domain. Interestingly, while both monomers of a homodimeric RING bind the E2 ligase, in heterodimeric RING, one of the monomers is active while the other is an adjuvant of the activity. However, there is no evidence of E2 cooperation in dimeric RINGs, as they bind very far apart from one another [10].

### 1.2. The HDAC Protein Family

The Histone DeAcetylase family is a group of enzymes that act as epigenetic modifiers or erasers that deacetylate lysine residues in histones, but also in other enzymes such as NF-kB, p53, HSP, and chaperones, amongst others [18,19]. The deacetylation of lysines drastically changes the charge of the protein and, in the case of histones, favors DNA packing [20]. 

The HDAC family is comprised of 18 types of proteins divided into four classes: Class I (HDAC1, HDAC2, HDAC3, and HDAC8), Class IIa (HDAC4, HDAC5, HDAC7, and HDAC9), Class IIb (HDAC6 and HDAC10), Class III (Sirt1-Sirt7), and Class IV (HDAC11). Of these four classes, all except Class III are metalloproteinases that rely on a zinc ion to catalyze the reaction. Class III members are called Sirtuins, and even if they fulfill the same cellular roles as the rest, their mechanism of action differs, as it depends on NAD^+^ [21]. 

Classes I, IIa, IIb, and IV are very similar in their catalytic domains, but they present key structural features that condition their catalytic activity and cellular localization. While Class I is predominantly found in the nucleus, Classes II and IV can move from the nucleus to the cytoplasm. The nuclear export of Class II is due to the presence of several serine residues at the *C*-terminal end of these HDACs that are susceptible of being phosphorylated by kinases [22]. On the other hand, the process by which this nuclear export is mediated in Class IV remains unknown [21].

The catalytic N-terminal domain is highly conserved among all Zn-dependent classes of HDAC, however Class IIa has a lower deacetylase activity than Class I [22]. Both Class I and Class II HDACs present a catalytic domain with a hydrophobic tunnel that leads to the catalytic Zn ion that is stabilized by the side chain of two aspartic acids and one histidine. Upon binding, the substrate is stabilized by the side chain of a tyrosine in the case of Class I and a histidine in the case of Class IIa and IIb. This change in amino acid is apparently the reason for the lower catalytic rate and the need of Class II HDACs of recruiting Class I HDACs to fulfill their catalytic activity [21]. Interestingly, Class IV HDACs (HDAC11), despite not presenting this amino acid change that affects substrate-binding, also present poor in vitro catalytic activity [20]. 

Selective HDAC inhibition is an interesting topic in drug discovery as HDACs are implicated in the development of many types of cancer, diabetes, and inflammatory, neurodegenerative, and cardiovascular diseases, amongst others [18,21,23,24,25]. Nonselective HDAC inhibitors (HDACi) or wide spectrum inhibitors (pan-HDACi) present many off target reactions and adverse effects [26] and pharmacokinetic problems [21]. However, despite the interest they have brought about, nowadays only inhibitors for HDAC6, SIRT1, and SIRT2 have been reported, which is indicative of the challenge the design of selective HDACi represents for the scientific community [27]. 

With the intention to overcome these problems and gain selectivity, in recent years a new drug design strategy has been explored to induce the selective degradation of undruggable proteins or proteins that are difficult to inhibit selectively. This new strategy involves the design of PROteolysis TArgeting Chimaeras (PROTACs).

### 1.3. PROteolysis TArgeting Chimaeras (PROTACs)

PROteolysis TArgeting Chimaeras (PROTACs) are large heterobifunctional molecules that present two specific moieties linked together by a flexible linker that may vary in length. A recent review by Petterson M. and Crews C.M. gives a clear overview of PROTAC development [28]. The first methionine aminopeptidase-2 (MetAp-2) directed PROTAC [29] was reported in 2001 and was very shortly followed by Estrogen Receptor and Androgen Receptor directed PROTACs in 2003 [30]. All of these PROTACs recruited the SCFβ-TRCP E3 ligase and needed to be directly injected in cells to show their degradation activity. Later on, the Von Hippel–Lindau E3 ligase was targeted with different small peptides to induce degradation of Aryl hydrocarbon Receptor Nuclear Translocator (ARNT) [31]. However, despite their ablity to induce proteasomal protein degradation, the use of a small peptide as E3 ligase recruiters was a drawback regarding bioavailability. This was overcome by the development of PROTACs which reicruited the E3 ligase using small organic molecules. The first example in this series was reported in 2008, where Vasilev L. et al. recruited the MDM2 E3 ligase using an MDM2 inhibitor [32]. 

These heterobifunctional molecules present a series of advantages that arise from their mechanism of action [33]. One of the moieties will recruit a specific E3 ligase, and the other one the protein of interest (POI) (Figure 1). When both proteins are recruited, the proximity between both will favor the ubiquitination of the POI that will then be recognized by the 26S proteasome. In the PROTAC structure, the moieties used to recruit the POI must present certain affinity for the POI, but a high selectivity and/or potency is not required. The selectivity of the degradation comes from the formation of a stable ternary complex E3:PROTAC:POI rather than from the POI recruiting moiety itself. However, the selection of an appropriate linker is crucial [34,35]. Additionally, due to this mechanism of action, a smaller dose of the PROTAC would be needed to produce the same effect as a conventional competitive inhibitor, thus reducing the possibilities of adverse effects when formulated as a medicine [36]. 

In the formation of ternary complexes, the “Hook effect” must be taken into account. This effect consists on the formation of a binary POI:PROTAC or PROTAC:E3 ligase complex when the concentration of the PROTAC is higher than that required for the ternary complex development. A key parameter to measure the stability of the POI:PROTAC:E3 complex is cooperativity (α). Positive cooperativity (α > 1) implies that the ternary complex formation is favored by the protein–protein interactions established between the POI and the E3 ligase. In the case of negative cooperativity (α < 1), this effect does not occur [28]. In addition, the selected linker also contributes to the positive cooperativity by establishing interactions with both proteins [37].

To date, many E3 ligases have been described to selectively ubiquitinate the different HDAC isoforms (Table 1). In this review, we attempt to gather functional and structural information on the E3 ligases and how HDACs are ubiquitinated. We also review the PROTACs that have been described to selectively favor that ubiquitination.

## 2. RING E3 Ligases

### 2.1. Cullin-RING Ligases

This family of RING ligases works by associating with several proteins to build a scaffold that will bring together the Ub-E2 ligase and the substrate protein: Cullin, the adaptor protein, the substrate recognition protein, the RING-box protein, and NEDD8.

The molecular scaffold in the middle of this association is the Cullin protein that belongs to a protein family, that in mammals, has eight family members (Cul1, Cul2, Cul3, Cul4A, Cul4B, Cul5, Cul7, and Cul9 (a.k.a PARC)). Cullins are elongated proteins that, at the *N*-terminal domain, bind to the adaptor protein, and at the *C*-terminal domain bind to the ubiquiting-like protein NEDD8 and the RING-box protein. This RING-box protein (Rbx), that can be found either as Rbx1 or Rbx2 and also as ROC1 and ROC2 (Regulator Of Cullin 1 and 2), presents a RING domain that can recruit the Ub-E2 ligase. Interestingly, the highly homologous (82% sequence identity) Cul4A and Cul4B bind to the same Rbx1 protein, however they target different protein substrates [39]. The substrate protein will bind to one of the more than 400 substrate-recognition proteins, that will, in their turn, be recruited by one of the four adaptor proteins (SKP1, ElonginB (EloB), ElonginC (EloC) and DDB1 (Damage DNA-Binding protein)) that will vary depending on the Cullin present in the Cullin-RING E3 scaffold [39,40,41]. 

**Figure 2:**
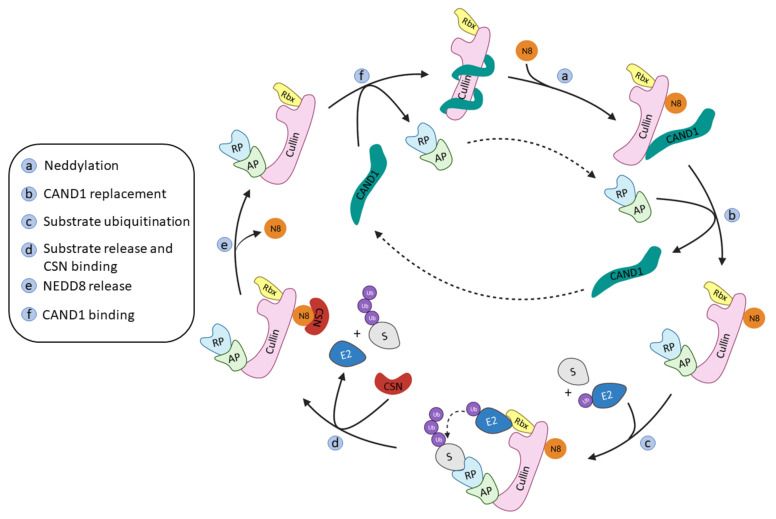
Schematic representation of the Cullin-RING ligases regulation and ubiquitination cycle adapted from Cui D. et al. [41].

The onset of the RING ligase ubiquitination cycle starts with the binding of NEDD8 to a lysine residue at the *C*-terminal end of Cullin, thus favoring the binding of the Ub-E2 ligase and an ulterior conformational change that will result in the ubiquitination of the substrate protein [15]. NEDD8 is removed from Cullin by the COP9 signalosome complex (CSN) and the substrate recognition/adaptor protein complex is removed by the binding of the regulating protein CAND1 (Cullin-Associated and Neddylation Dissociated 1). The binding of a new NEDD8 protein to Cullin will destabilize the binding of CAND1 and restart the whole process again (Figure 2) [41]. 

#### 2.1.1. Cul3-KCTD

The KCTD (Potassium Channel Tetramerization Domain) family is made up of 25 members (KCTD1–21, TNFAIP1, BTBD10, KCNRG, and SHKBP1) that can be phylogenetically grouped into seven different subgroups [42]. The name of this family arises from the high structural homology with the BTB (bric-à-brac, tramtrack, broad complex) domain of voltage-dependent potassium channels [43]. These proteins present a well conserved BTB N-terminal domain and a variable *C*-terminal domain. The variability of this domain is necessary as it is in charge of the recognition of different proteins [44]. Several KCTD family members (KCTD2, 5, 6, 10, 11, 13, 17, 21, and TNFAIP1) have been reported to bind to Cullin-3 and act as substrate-adaptor proteins [43], of which, KCTD6, KCTD11, and KCTD21 are known HDAC1 adaptors [45].

**Figure 3:**
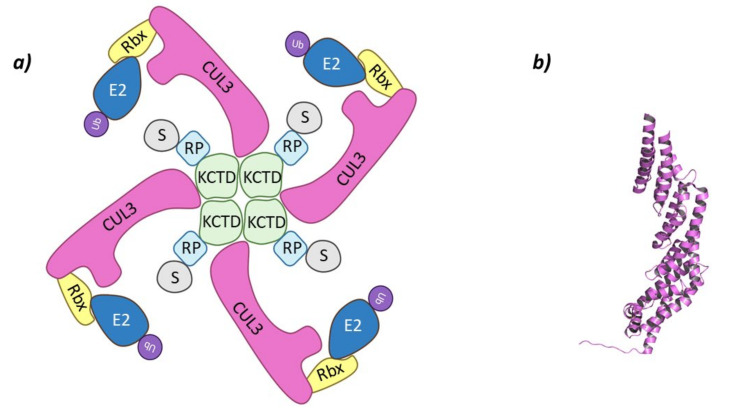
Cul3-KCTD. (**a**) Schematic representation of the assembly of the Cul3-KCTD E3 ligase adapted from Correale S. et al. [46]: adaptor protein KCTD in green, Recognition Protein (RP) in light blue, Substrate protein (S) in grey, Cullin-3 (Cul3) in magenta, Ring Box Protein (Rbx) in yellow, E2 ligase (E2) in dark blue, and ubiquitin (Ub) in purple. (**b**) 3D representation of the incomplete Cullin-3 as found in PDB code 4AP2.

These Cullin-RING E3 ligases complexes are formed by Cullin-3 that binds Rbx1 at the *C*-terminal end to recruit de Ub-E2 ligase, while at the *N*-terminal domain binds the substrate adaptor containing BTB domain, KCTD6, KCTD11, or KCTD21 (Figure 3) [44,45]. 

These three adaptor proteins were englobed by De Smaele E. et al. in a subfamily called KCASH (KCTD containing, Cullin-3 adaptor, suppressor of Hedgehog), and the members carry the following names KCASH1 (KCTD11), KCASH2 (KCTD21), and KCASH3 (KCTD6). The RENKCTD11(KCASH1) protein is encoded by the tumor suppressor gene KCTD11REN that is located in the chromosome 17p13 along with other tumor suppressing genes [47]. KCASH2 and KCASH3 are encoded by bi-exonic genes located in chromosomes 11q14 and 3p14, respectively; and, similar to KCASH1, they are found either absent or methylated (except for KCASH3) in cancer [45]. It has been seen that these three KCASH proteins present reduced expression in medulloblastoma associated to an oncogenic Hedgehog signaling pathway [48]. The activation of the Hedgehog pathway promotes the activation of Gli 1 and 2 (glioma-associated proteins) that are transcription factors that will, in their turn, regulate gene expression [48]. Research carried out by Canettieri G. et al. determined that the Gli1 and Gli2 are themselves regulated by acetylation and deacetylation, and that Cul3-REN KCTD11 forms a supramolecular complex with HDAC1 that participates in the regulation of this pathway. The HDAC1 mediated activation of Gli 1 and 2 is counteracted by the Cul3-REN KCTD11 system that will favor the deactivation by ubiquitination of HDAC1 [49]. The similar study carried out by De Smaele E. et al. demonstrated that KCASH2 and KCASH3 act through an analogous mechanism [45]. Moreover, the RENKCTD11 protein has been found to present a reduced expression in prostate adenocarcinoma [50], whereas the expression of the sAnk1.5/KCTD6/Cul3 complex in knockout mice seems to be implicated in the development of hereditary spherocytosis [51].

The level of structural organization amongst the KCTD family is variable, as these family members tend to form oligomers of different degrees of complexity by the interaction of their BTB domains. While there is no structural information on the formation of oligomers of KCTD21, both KCTD11 and KCTD6 usually form tetramers in solution [42,46], and recently electron microscopy has demonstrated that KCTD11 can also be found as a pentamer [52]. In addition, KCTD11/KCTD6 and KCTD11/KCTD21 heterooligomers have been identified, whereas KCTD6/KCTD21 heterooligomers have not been described to date [45]. The interaction of the BTB domain with Cullin-3 has been defined through a combination of both experimental and computational methods. It has been observed that KCTD protein binds to Cullin-3 anoligomeric form, as the recruitment of Cullin-3 by the BTB domain is through α-helixes H2 and H5 of the *N*-terminal domain, which interact with the BTB domains of two adjacent monomers of the adaptor proteins [42,44,45].

Co-immuno precipitation assays carried out by De Smaele E. et al. have demonstrated that both KCTD11 and KCTD21 promote the ubiquitination of HDAC1, whereas KCTD6 needs to recruit KCTD11 [45].

#### 2.1.2. CRL2^VHL^

The von Hippel–Lindau tumor suppressor protein (pVHL) is the substrate recognition protein of the Cul2-Rbx1-EloBC-VHL E3 ligase system. This protein is named after the von Hippel–Lindau inherited cancer syndrome and presents two domains: one implicated in the nuclear export of the CRL2^VHL^ complex that, when mutated, leads to the onset of cancer; and another responsible for the binding of substrate proteins [53]. One of the most well known ubiquitination substrates of pVHL is the Hypoxia Inducible Factor 1 (HIF-1) implicated in the expression of genes involved in angiogenesis and erythropoiesis [54,55,56]. This transcription factor is naturally regulated by prolyl hydroxylases (PHDs) that, by hydroxylating proline residues, mark HIF-1 for degradation by the E3 ligase CRL2^VHL^. Most recently, some studies have shown that CRL2^VHL^ t is also implicated in the regulation of the EGFR, Sprouty2 proteins, and the HIF1α-related p97 [57,58,59]. 

Interestingly, CRL2^VHL^ can be hijacked by PROTACs to achieve the proteasomal degradation of non-natural substrates of this E3 ligase [60,61]. 

**Figure 4:**
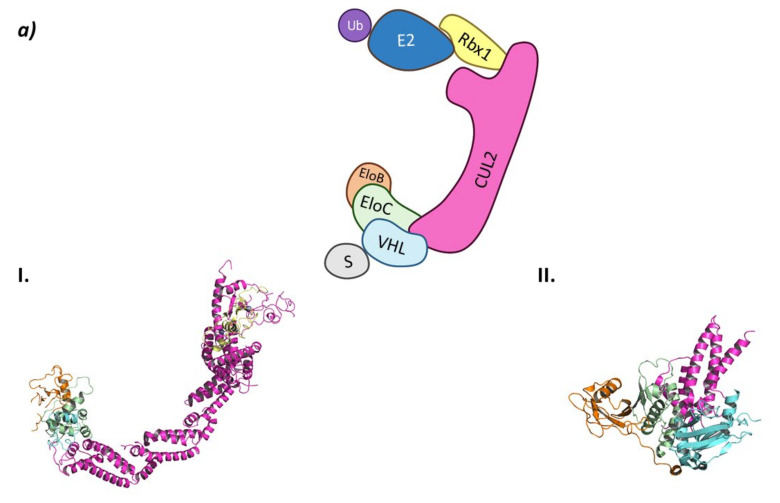

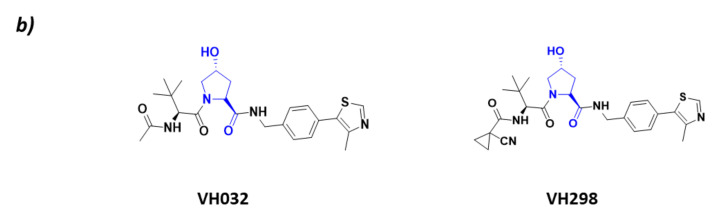
CRL2^VHL^ (**a**) Schematic representation of the assembly of the CRL2^VHL^ E3 ligase: ubiquitin (Ub) in purple, E2 ligase (E2) in dark blue, Ring Box Protein 1 (Rbx1) in yellow, Cullin-2 (Cul2) in magenta, adaptor protein Elongin B (EloB) in orange and Elongin C (EloC) in green, substrate recognition protein VHL (VHL) in light blue, and substrate protein in grey. Protein assembly I is the 3D representation of the system made up of Cul2, EloB, EloC, and a small α-helix of VHL as found in PDB code 5N4W. Protein assembly II is the 3D representation of the *N*-terminal end of Cul2 bound to EloB, EloC, and VHL, as found in PDB code 4WQO. (**b**) 2D representation of the structures of the main VHL:HIF-1α inhibitors developed by Ciulli A. et al. [63,64]. The hydroxylated proline that mimics the activated HIF-1 α is represented in blue.

In the CRL2^VHL^ system, Cullin-2 recruits Rbx1 at the *C*-terminal end while heterodimeric adaptor proteins called ElonginB (EloB) and ElonginC (EloC) bind to the *N*-terminal end of Cullin-2. Of this heterodimeric adaptor, EloC interacts with Cullin-2 through two α-helixes, while EloB apparently stabilizes the EloC conformation by interacting in opposition to Cullin-2. The interaction of EloC with Cullin-2 creates a surface where the substrate recognition protein pVHL binds (Figure 4) [62]. 

As HIF-1 is one of the main CRL2^VHL^ substrates, many inhibitors of the interaction of HIF with this ligase have been described with the aim of becoming antiangiogenic drug candidates. All these inhibitors bind to the substrate recognition protein pVHL, thus impairing its binding to HIF [63,65]. The main VHL inhibitors, VH032 and VH298 (Figure 4), have been developed by the research group of Alessio Ciulli. From a structural point of view, these inhibitors mimic the structure of the hydroxylated proline present in HIF-1 after it has been activated by PHDs [63,64]. 

#### 2.1.3. CRL4^CRBN^

**Figure 5:**
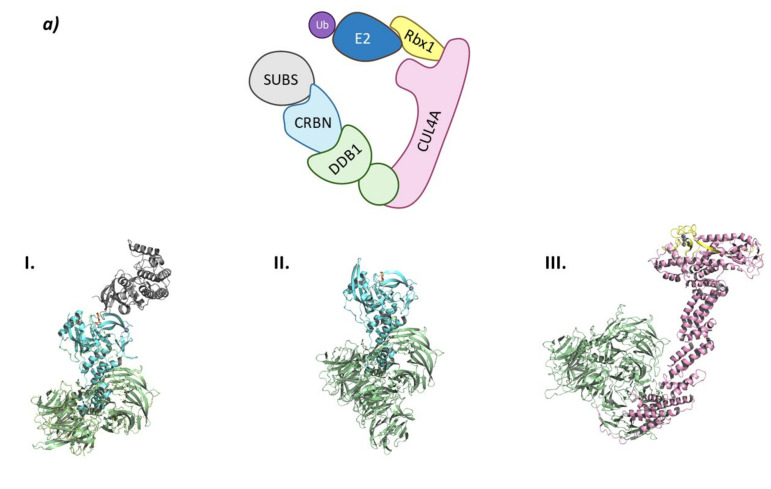

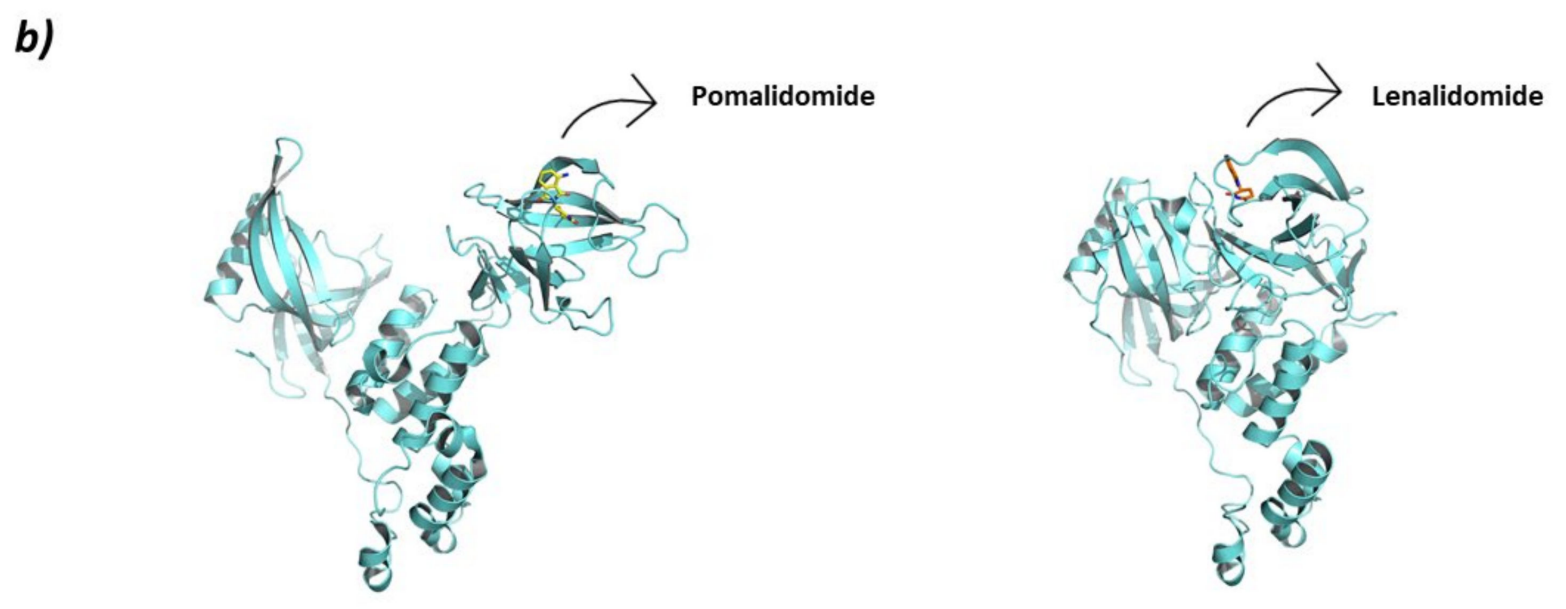
CRL4^CRBN^ (**a**) Schematic representation of the assembly of the CRL4^CRBN^ E3 ligase: ubiquitin (Ub) in purple, E2 ligase (E2) in dark blue, Ring Box Protein 1 (Rbx1) in yellow, Cullin-4A (Cul4A) in magenta, adaptor protein DDB1 (DDB1) in green, substrate recognition protein CRBN (CRBN) in light blue, and substrate protein in grey. Protein assembly I is the 3D representation of the system made up of a fragment of DDB1, CRBN bound to lenalidomide (orange sticks) and the substrate protein CK1α as found in PDB code 5FQD. Protein assembly II is the 3D representation of the full sequence of DDB1, CRBN bound to lenalidomide (orange sticks) as found in PDB code 4CI2. Protein assembly III is the 3D representation of the Rbx1, Cul4A, and DDB1 as found in PDB code 2HYE. (**b**) 3D representation of the two different conformations adopted by CRBN. Open conformation when bound to Ikaros as found in PDB code 6H0F (left), and closed conformation when bound to CK1α as found in PDB code 5FQD (right). For the sake of clarity, neither the fragment of Ikaros nor the full CK1α protein bound to CRBN present in the PDB codes are shown.

This E3 ligase complex is formed by the association of Cullin-4 to Rbx1 and NEDD8 to form the Ub-E2 recruiting site at the *C*-terminal end, and of the adaptor protein DDB1 (Damage DNA-Binding protein 1) and CRBN as the substrate recruiting unit. (Figure 5) [66]. 

CRBN can be located in the nucleus and cytoplasm, and is implicated in many cellular processes. It impairs the phosphorylation/activation of AMPK by binding to the catalytic α subunit blocking the energetic metabolism carried out by this kinase [66]. CRBN has also been found to be the genetic cause of an autosomal recessive non-syndromic mental retardation related to both an anomaly in the CRBN locus of the 3p chromosome and the interaction of CRBN with the large-conductance calcium-activated potassium (BKCA) channel [66,67,68]. Additionally, it has been demonstrated that CRBN is able to ubiquitinate other ion channels, such as the CLC-2 voltage-dependent chlorine channels [69]. 

**Figure 6:**
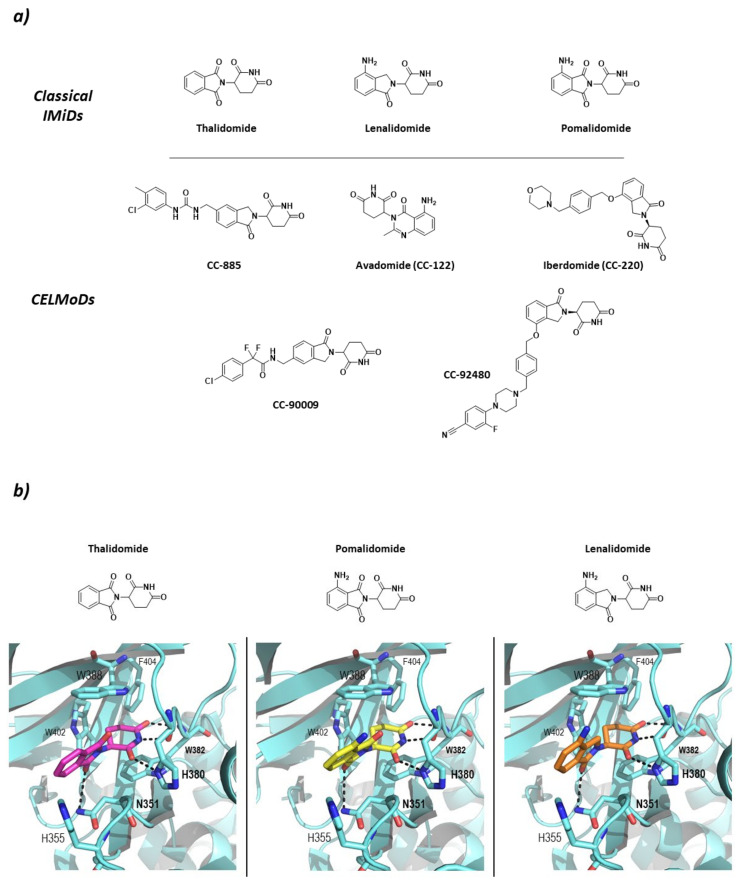
Classical ImiDs and CELMoDs (**a**) 2D representation of the structures of the Classical ImiDs and CELMoDs reported to date that have reached clinical trials. (**b**) 3D representation of the binding mode to CRBN (blue) of the classical ImiDs: thalidomide (magenta), pomalidomide (yellow), and lenalidomide (orange) as found in PDB codes 4CI1, 4CI3, and 4CI2, respectively. Hydrogen bonds are shown as dashed lines.

Studies carried out in zebra fish have reported that CRBN is responsible for limb growth, which, linked to the fact that ImmunoModulatory Drugs (IMiDs) such as thalidomide and derivatives lenalidomide and pomalidomide bind to it and work as “molecular glues” (Figure 6), points in the direction of the reason for the well-known teratogenicity shown by thalidomide at the molecular level [70]. However, the binding of IMiDs has been found to alter the activity of the CRL4^CRBN^ system by increasing the affinity for proteins that are not normal substrates of this RING E3 ligase. Additionally, IMiDs have been found not only to have immunomodulative effects on T cells (hence the name), but also to have antiproliferative effects on multiple myeloma cell types by recruiting the transcription factors Ikaros (IKZF1) and Aiolos (IKZF3) for its degradation [66]. It has also been found that the IMiD-bound CRL4^CRBN^ system is able to ubiquitinate other C2H2 Zn-finger proteins [71], glutamine synthetase enzyme [72], and the serine/threonine protein kinase CK1 [73]. Additionally, with the use of PROTACs, the CRL4^CRBN^ mediated degradation of non-natural substrates such as CK2 [74] and members of Classes I, II, and III HDACs [75], amongst others, has been achieved. More recently, classical IMiD related compounds have been described as Cereblon Modulators (CELMoDs) (Figure 6) as they fulfill the same substrate-recruiting and CRBN-dependent degradation function as thalidomide, lenalidomide, and pomalidomide. Most of these compounds are in the preclinical phases of development [76,77,78,79,80], but some have already reached clinical trials for the treatment of different types of cancer and autoimmune diseases, such as Avadomide (CC-122) and Iberdomide (CC-220). Avadomide is in Phase I clinical trials for the treatment of diffuse large B-cell (DLBCL) and follicular lymphoma [81], as well as for the treatment of advanced stages of cancer [82]. Iberdomide has entered Phase II clinical trials for the treatment of Lupus and Phase Ib/IIa for the treatment of relapsed refractory multiple myeloma [83].

CRBN has been classified as an abnormal type of DCAF (DDB1-Cul4 Associated Factor) that does not present the typical WD-repeat domain of DCAF proteins, but specifically binds to DDB1, which is known to recognize DCAF proteins. The structure of CRBN can be divided into two domains: *N*-terminal domain or Lon-like Domain (LLD); and the *C*-terminal domain or Thalidomide-binding domain where the Thalidomide Binding Site (TBS) is located. The core of the *C*-terminal domain is made up of six antiparallel β-sheets that coordinate a structural Zn ion. The TBS is a very small hydrophobic pocket, located in the surface of CRBN opposite to the DDB1-binding domain, formed by the side chains of three tryptophan residues that make the walls and a phenylalanine that makes the bottom of the pocket (Trp380, Trp386, Trp400, and Phe402; numbering from UniProtkb code Q96SW2). The IMiDs insert the glutarimide moiety in the small pocket and stabilize the binding by two hydrogen bonds with the backbones of His378 and Trp380 and one with the side chain of His378. In addition, the isoindolinone ring stablishes a hydrogen bond with the side chain of N351 (Figure 6) [66,84].

Interestingly, depending on the protein substrate, the IMiD-bound CRL4^CRBN^ system can adopt different conformations. The conformational change undergone by CRBN is reminiscent of the opening and closing movement of the thumb: closed when it is near the hand and opened when it is away from the hand. When CRBN interacts with Ikaros, the conformation is opened (PDB code 6H0F), but when it interacts with CK1 the conformation is closed (PDB code 5FQD) (Figure 5) [71,84]. 

#### 2.1.4. Cul3^SPOP^

**Figure 7:**
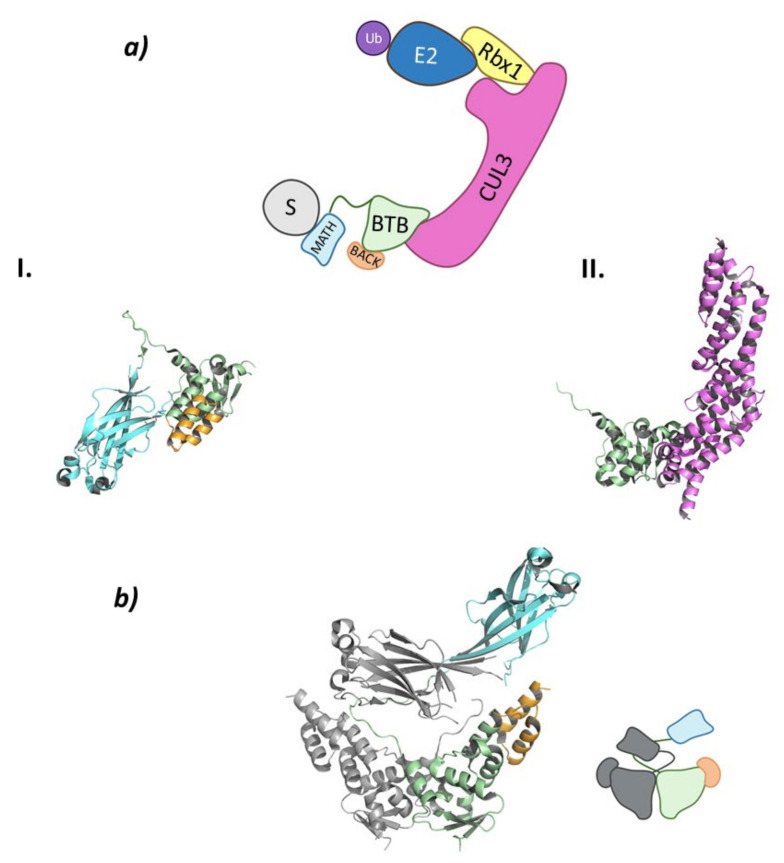
Cul3^SPOP^ (**a**) Schematic representation of the assembly of the Cul3-SPOP E3 ligase: ubiquitin (Ub) in purple, E2 ligase (E2) in dark blue, Ring Box Protein 1 (Rbx1) in yellow, Cullin-3 (Cul3) in magenta, substrate protein (S) in grey, and adaptor protein SPOP shown as the three domains: MATH in light blue, BTB in green, and BACK in orange. Protein assembly I is the PyMOL cartoon representation of the three domains that make up SPOP as found in PDB code 3HDI. Protein assembly II is the 3D representation of the fragment of Cul3 bound to the BTB domain as found in PDB code 4EOZ. (**b**) 3D representation of the dimerization of SPOP as found in PDB code 3HDI.

The Cullin-3 protein that works as the scaffold of this system interacts with Rbx1 at the *C*-terminal end and the SPOP protein at the *N*-terminal end. The SPOP protein, whose name stands for speckle-type POZ proteins due to the dotted pattern it produced in the nucleus of the cells, is the substrate-recognition protein of this Cullin-RING E3 ligase complex that also belongs to the BTB (bric-à-brac, tramtrack, broad complex) family (Figure 7) [85,86].

Due to the nuclear residence of SPOP, it recruits as substrates different nuclear proteins that are at the end of many cellular pathways. Works carried out by Cuneo M. J. et al. listed a total of 26 SPOP substrates that ranged from nuclear receptors, epigenetic modifiers, transcription factors, and cell cycle modulators, to nuclear phosphatases [85]. Substrate-recognition by SPOP can be altered by different types of inhibitors, however no compound has yet reached clinical trials. In 2016, an inhibitor of the SPOP-substrate interaction was designed and synthesised by Qiang G. Z. et al. leading to the removal of SPOP from the “undruggable” proteome. This compound inhibits the ubiquitination and degradation of tumor suppressors PTEN and DUSP7 whose conjoint absence added to the overexpression of SPOP in the cytoplasm is linked to kidney cancer [87]. In this line, research carried out by Tan Y. et al. has identified HDAC6 as a ubiquitination target of this Cullin-RING E3 ligase, by means of two different inhibitors (proteasome and E3 ligase inhibitor) [88]. 

SPOP can be divided into three domains: a Meprin and TRAF-C Homology (MATH) domain at the *N*-terminal end; the BTB domain in the center part of the protein; and a *C*-terminal BTB and *C*-terminal Kelch (BACK) domain (Figure 7). This last domain is responsible for the nuclear localization [85,89]. The MATH domain is responsible for substrate binding through one of the exterior antiparallel β-sheets that make up the structure. This domain specifically recognizes proteins that present one or multiple specific, highly conserved amino acid sequences. These sequences, known as SPOP-binding (SB) sequences, are rich in serine and threonine and present in the first and second positions a non-polar and a polar amino acid, respectively [85]. The central BTB domain is not only responsible for interacting with Cullin-3 but is also involved in the dimerization of this ligase between the BTB domains of two Cul3^SPOP^ (Figure 7). In addition, this E3 ligase is able to oligomerize by simultaneous interaction between the BTB and BACK domains of different Cul3^SPOP^ [90]. When forming dimers or oligomers, the specificity for the substrate and the catalytic activity of this ligase are increased, as two Cul3^SPOP^ monomers can recognize two different SB sequences of the same target protein [85,91]. The interaction between the BTB domains mainly occurs for the formation of homodimeric SPOP or heterodimers formed by SPOP and its homologous, SPOP-like (SPOPL) ligase. Nevertheless, heterodimeric combinations may occur, but they result in a decrease in the E3 ligase activity of Cul3^SPOP^ [91]. The *C*-terminal BACK domain is made up of five α-helixes and is located near the BTB dimerization site. Linear oligomers that potentiate the activity of the ligase can be formed by simultaneous interaction of the BTB and BACK domains [89,90]. 

### 2.2. Monomeric RING

#### 2.2.1. CHFR

The integrity of the genetic material is ensured during cell cycle by different checkpoints that work at the different stages of cell division; therefore, mutation in the genes that encode the effectors of these checkpoints is usually found in different types of cancer. Amongst these checkpoint effectors, CHFR (Checkpoint Protein with FHA and RING finger domains) has been shown to regulate the entrance into the metaphase of cell division as it delays chromosome condensation and is considered to be a tumor suppressor [92,93]. CHFR is located in the nucleus and is known to regulate the acetylation levels of different proteins by regulating the activity of SIRT1, a non-Zn dependent HDAC [94]. Additionally, Oh Y. M. et al. demonstrated that cancer cell lines where CHFR was missing were reverted by forcing the expression of CHFR through the downregulation of many of the substrates of this ligase, HDAC1 being one of them [95].

**Figure 8:**
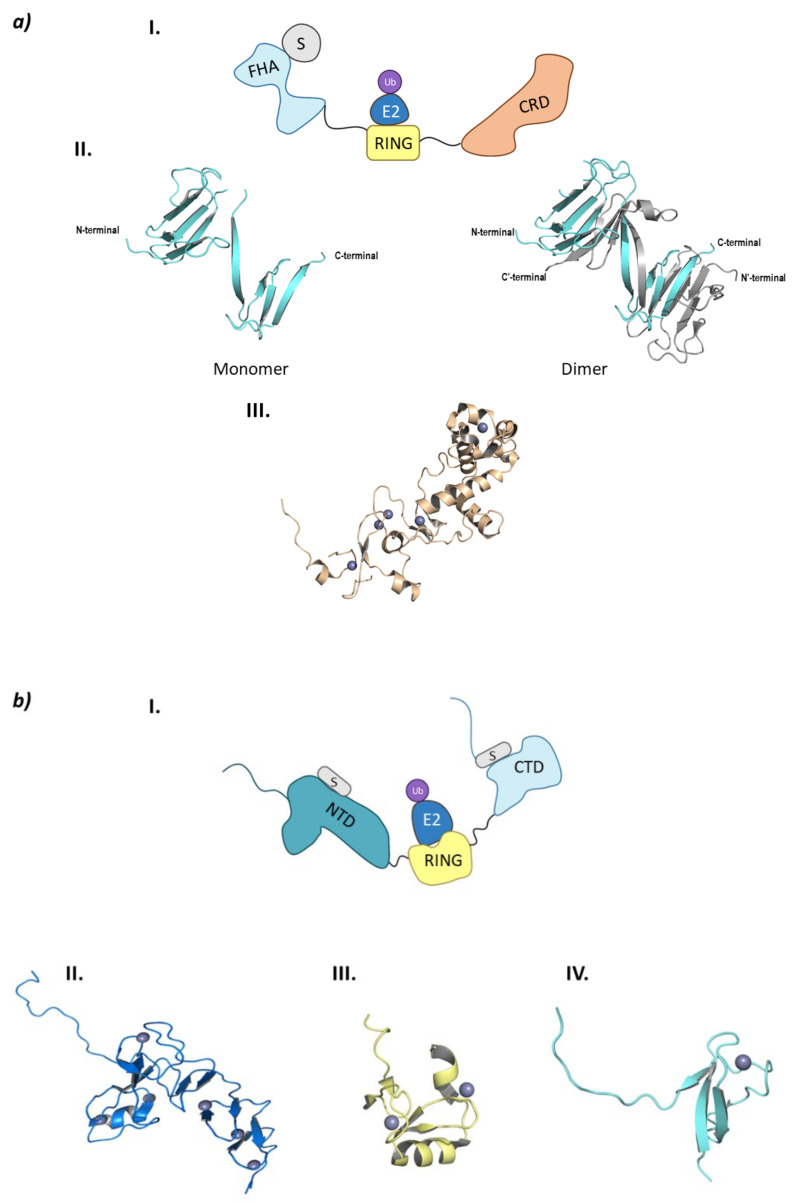

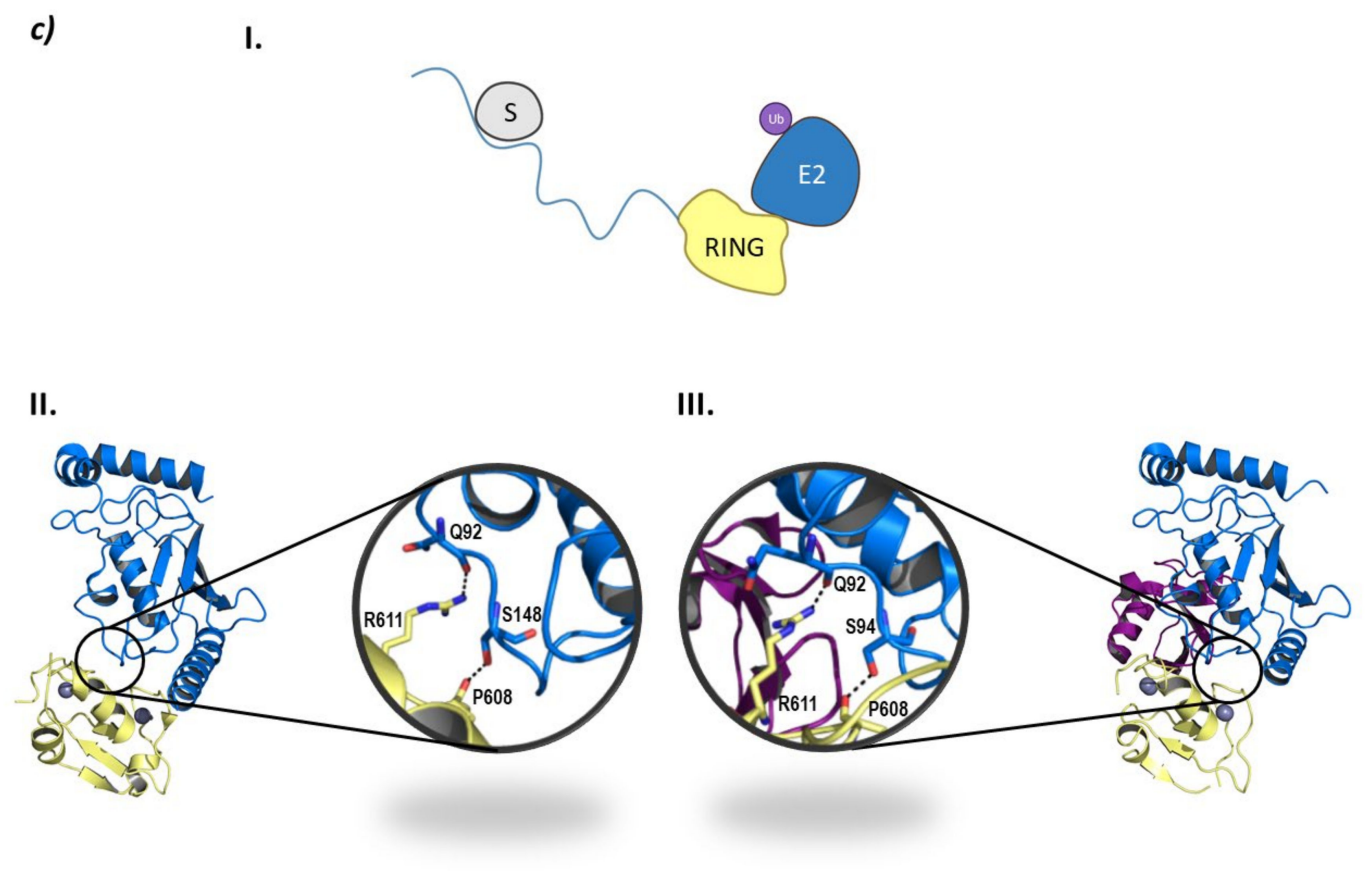
Monomeric RING E3 ligases. (**a**) CHFR E3 ligase. I is the schematic representation of the assembly of the CHFR E3 ligase: ubiquitin (Ub) in purple, E2 ligase (E2) in dark blue, Ring domain (RING) in yellow, substrate recognition domain FHA (FHA) in light blue, substrate protein in grey, and the cysteine rich domain (CRD) in orange. Protein assembly II is the 3D representation of the monomeric and dimeric conformations of FHA as found in PDB code 1LGQ. Protein assembly III is the 3D representation of the CRD domain as found in PDB code 2XP0. (**b**) PIRH2 E3 ligase. I is the Schematic representation of the assembly of the PIRH2 E3 ligase: ubiquitin (Ub) in purple, E2 ligase (E2) in dark blue, Ring domain (RING) in yellow, the *N*-terminal domain (NTD) in turquoise, the *C*-termina domain (CTD) in light blue, and the substrate protein in grey. Protein assembly II is the 3D representation of the NTD as found in PDB code 2K2C. Protein assembly III is the 3D representation of the RING domain as found in PDB code 2JRJ. Protein assembly IV is the 3D representation of the CTD as found in PDB code 2K2D. (**c**) RLIM E3 ligase. I is the Schematic representation of the assembly of the RLIM E3 ligase: ubiquitin (Ub) in purple, E2 ligase (E2) in dark blue, Ring domain (RING) in yellow, the disordered *N*-terminal end in light blue, and the substrate protein in grey. Protein assembly II is the 3D representation of the RING domain bound to the E2 ligase Ube2e2 as found in PDB code 6W9A. Protein assembly III is the 3D representation of the RING domain bound to the E2 ligase Ube2d2 as found in PDB code 6W9D. Hydrogen bonds are shown as dashed lines and zinc ions as grey spheres.

CHFR is made up of three domains: FHA (ForkHead-Associated) domain at the *N*-terminal end, the RING domain, and the CRD (Cystein-Rich Domain) at the *C*-terminal end (Figure 8a).

The FHA domain is in charge of interacting with phosphorylated proteins and has a β-sandwich structure made up of ten β-sheets and a small α-helix inserted between sheets 6 and 7 [96]. Despite being crystalized (PDB codes 1LGQ and 1LGP), the structure is not fully characterized, as in the crystal structure 1LGQ; it appears to be a segment-swapped dimer, but it is not clear whether this domain can dimerize or it is an artefact of the crystal structure (Figure 8) [97]. Interestingly, in 2002 a CHFR homologue (Chf2) was found to oligomerize in response to DNA damage, which raises questions of whether CHFR could have the same behavior [98]. The CRD binds five Zn^2+^ ions located in four Zn-binding domains, of which the last conforms the PAR-Binding Zinc finger (PBZ). This PBZ is the binding site for PolyADP-ribose (PAR) that constitutes a post-translational modification that rises from DNA damage. Despite not being crystalized in complex with CHFR, Oberoi J. et al. were able to crystalize the CRD of CHFR in complex with PAR-structurally related ligands [99]. As for the E3 ligase and checkpoint-responsible RING domain, no crystal structures have been obtained to date.

#### 2.2.2. PIRH2 (RCHY1)

PirH2 (p53-induced protein with a RING-H2 domain) is an E3 ligase that has not been fully structurally characterized, and that presents a tissue-dependent role in the regulation of tumoral processes, as it is shown to be overexpressed in different types of cancers, such as lung and prostate cancer, but it is under expressed in other types of cancers such as breast and ovarian cancer [100]. These two expression profiles can be explained according to the main protein substrates of this ligase, as some are oncoproteins as *c*-Myc, and others are oncosuppresors (p53 and homologous proteins p63 and p73, p27, Chk2, Androgen Receptor (AR), HDAC1 and HDAC2, and polH) [100,101,102,103,104]. Apart from ubiquitinating itself, PirH2 can ubiquitinate the tetrameric (active) form of p53, thus targeting it for proteasomal degradation [101,103]. PirH2 is also able to mediate in the proteasomal degradation of the structural homologues p63 and p73, which are involved in the proliferation and differentiation of epidermic cells and in cellular processes related with neurological development, respectively [102]. On the other hand, some head and neck cancer cell lines present an inverse correlation between the levels of the tumor suppressor p27 and PirH2 [105]. Another PirH2 substrate is the Chk2 kinase that is in charge of phosphorylating serine and threonine residues in response to DNA damage, as well as acting as a cell cycle checkpoint. Mutations on the Chk2 gene are related with the occurrence of different types of cancers such as lung and prostate cancers. The stability of this kinase is determined by the phosphorylation profile, as the phosphorylation of Ser460 protects it from proteasomal degradation; but interestingly, when Ser460 is mutated to alanine, Chk2 is ubiquitinated by PirH2 and subsequently degraded by the proteasome [102,106]. PirH2 is also related to the development of prostate cancer as it interacts with the AR as well as with one of the AR repressors, HDAC1 [103]. Additionally, a meta analysis carried out by Choi M. et al. in lung and colon cell lines proved that PirH2 is also responsible for the ubiquitination of HDAC2 [104]. On the other hand, PirH2 can ubiquitinate the oncoprotein *c*-Myc which has been related to an increase in the proliferation of blood cells (T and B-type lymphocytes, amongst others) [100,102]. A study carried out by Yang L. et al. on multiple myeloma cell lines associated the development of resistance to treatment with bortezomib with the overexpression of PirH2, and the consequent reduction in a downstream protein in the signaling route of NF-kB [107]. 

PirH2 can be found in different isoforms (A, B, C, C’, and D). PirH2A is the only isoform that presents E3 ligase activity and the full 261 amino acid sequence. This isoform is formed by an *N*-terminal domain or CHY-Zn-finger domain (also responsible for the other name of this ligase: RCHY1 (RING-finger and CHY-zinc-finger domain-containing protein 1), a RING-H2 (C3H2C3) central domain and a terminal *C*-terminal domain (Figure 8b) [100]. 

Along the three domains, PirH2 presents nine Zn^2+^ ions coordinated with cysteine and histidine residues: six located in the *N*-terminal domain, two in the RING domain and one in the *C*-terminal domain. The *N*-terminal domain is formed by the N and C lobe perpendicular to one another, that are made up of small antiparallel β-sheets and a α-helix. This *N*-terminal domain is able to weakly interact with the DNA-binding domain (DBD) of p53, thus contributing to the ubiquitination of this tumor suppressor. On the other side, the *C*-terminal domain presents a disordered *N*-terminal end, whereas the *C*-terminal end presents a small globular structure near the last zinc ion. Additionally, residues Ala249 and Ile256 create an interacting surface with the p53 tetramerization domain (TET) [101,108]. Unlike the interaction between the *N*-terminal domain and p53, that of the *C*-terminal domain and the p53 tetramerization domain is crucial for the ubiquitination and degradation of p53. To date, there is no crystal structure of the full assembly of this E3 ligase, but there are crystal structures of the different separate domains. However, Sheng Y. et al. proposed an interaction model of the three domains where they determined that a certain flexibility should exist between the RING and *C*-terminal domain in order to explain the simultaneous interaction of the ubiquitin-bound E2 ligase and the p53 protein with PirH2 [101].

#### 2.2.3. RLIM (RNF12)

Bach I. et al. were the first to identify RLIM (RING finger LIM domain-binding protein) also known as RNF12 (RING finger 12) [109,110]. They determined that the interaction of RLIM with LIM domain-containing transcription factors (such as Lhx2 or Lhx3) promote the recruitment of co-repressor complexes, whereas the LIM cofactors (CLIM) are ubiquitinated by this E3 ligase and subsequently degraded [109,111]. Additionally, RLIM has an important role in neurological development by triggering the inactivation of the X chromosome in early stages of development by ubiquitination of Rex1 [110]. In fact, a mutation of this E3 ligase has been associated with the occurrence of a subtype of intellectual disability named XLID (X-Linked Intellectual Disability) [112]. RLIM also acts as an activator of the signaling pathway of TGF-β by degrading Smad7. Smad7 is an inhibitor of the TGF-β signaling pathway by recruiting the HECT E3 ligase Smurf2 so that it will degrade the specific receptors of the TGF-β [113]. Another of the substrate proteins of RLIM is HDAC2. Interestingly, apart from an antipsychotic effect, valproic acid acts as a Class I HDAC inhibitor as well as inducing selective degradation of HDAC2. This inhibitor induces the expression of the E2 UbcH8 and, even if it does not affect the levels of RLIM, valproic acid is able to induce the degradation of HDAC2 by a still unknown mechanism [114]. On the other hand, RLIM is able to polyubiquitinate proteins without targeting them for degradation by the 26S proteasome. Gao R. et al. reported that RLIM diminished the transcription activity of *c*-Myc by ubiquitination. However, they were able to demonstrate it for only two of the target genes E2F2 and Nucleolin [115]. Additionally, RLIM takes part in an indirect fashion in the regulation of the levels of p53. On the one hand, it ubiquitinates MDM2 that regulates this tumor suppressor; and, on the other hand, levels of p53 reduce the expression of RLIM [116]. As in other cases, RLIM levels are regulated by autoubiquitination and by other E3 ligases such as SiAH1/2 and TRIM28 [102,108,109] TRIM28 can be overexpressed in lung cancer, which translates to a reduction in the RLIM levels and, as a consequence, in an increased degradation of p53 mediated by MDM2 [117]. 

RLIM is a monomeric E3 ligase made up of 624 amino acids which are structurally disordered, and contains only one defined RING domain in the *C*-terminal end (Figure 8c) [118]. RLIM is mainly located in the nucleus of the cell; however, with the phosphorylation of a serine residue in a nuclear export signal outside the RING domain, it can be exported to the cytoplasm. The RLIM orthologue in mice (Rlim/RNF12) presents a Basic Domain (BD) that is in charge of substrate recognition [110]. Additionally, RLIM presents very few lysine residues, which could account for a resistance to ubiquitination. Middleton J.A. et al. identified Ube2d2 and Ube2e2 as two E2 ligases capable of interacting with RLIM [118]. RLIM establishes hydrophobic interactions with both of them, as well as hydrogen bonds between Arg611 and Pro608 in RLIM, and Gln92 and Ser94/Ser148 in Ube2d2 and Ube2e2, respectively (Figure 8).

Despite the E2-Ub complex presenting a closed conformation with respect to RLIM, no typical interaction of Arg611 and ubiquitin can be found [118]. To date, only the RING domain of RLIM has been crystalized bound to the E2 ligases Ube2d2 (PDB code 6W7Z) and Ube2e2 (PDB code 6W9A), as well as to the Ube2d2-Ub complex (PDB code 6W9D) (Figure 8). Despite the presence of a RING domain dimer in crystal structure 6W9A, Middleton J. A et al. experimentally determined that the *C*-terminal domain that contains RING does not dimerize [118]. 

### 2.3. Homodimeric RING

#### SIAH2

The SIAH (Seven in Absentia Homolog) family in humans is made up of three members: SIAH1, SIAH2, and SIAH3. SIAH2 is regulated through the phosphorylation of many kinases that are involved in hypoxia [119]. SIAH2 regulates the cellular response to hypoxia through an intricate mechanism. In normal oxygen conditions, HIF-1 is targeted for VHL ubiquitination by the PHD-mediated hydroxylation of prolines. However, in hypoxia conditions, the SIAH2 degrades PHDs, which results in an increased life span of HIF-1 which, non hydroxylated, is not recognized by VHL [119]. Additionally, the HIF-1 levels can also be regulated by the SIAH2-mediated degradation of LATS2 (downstream kinase of the Hippo signaling pathway) [120]. SIAH2 is also involved in the expression of antioxidant proteins as a response to ROS (Reactive Oxygen Species) [121]. 

SIAH2 also has a role in the immune response, as it acts as a checkpoint protein in regulatory T-cells, which is important in cancer development. Scortegagna M. et al. determined that treatment of SIAH2 double knockout mice with melanoma cells resulted in an increased immune response and inhibition of tumor growth due to a pronounced reduction in regulatory T-cell levels [122]. 

The E3 ligase activity of SIAH2 is inhibited by the nuclear proto-oncogene Ski protein, which leads to an increase in the nuclear levels of HDAC3. This is a clear indicator of the degradation of HDAC3 by this E3 ligase [123]. Additionally, Zhang Y. et al. described the relationship that exists between SIAH2, HDAC3, PIWIL2, and CK2α. PIWIL2 (PIWI-Like protein 2) is an endoribonuclease that reduces the probability of ubiquitination of HDAC3 by SIAH2 and favors the activation by phosphorylation of HDAC3 by CK2α. Interestingly, in the context of cancer, a co-expression of PIWIL2 and HDAC3 translates in an increased proliferation rate and reduced p53-mediated apoptosis [123].

**Figure 9:**
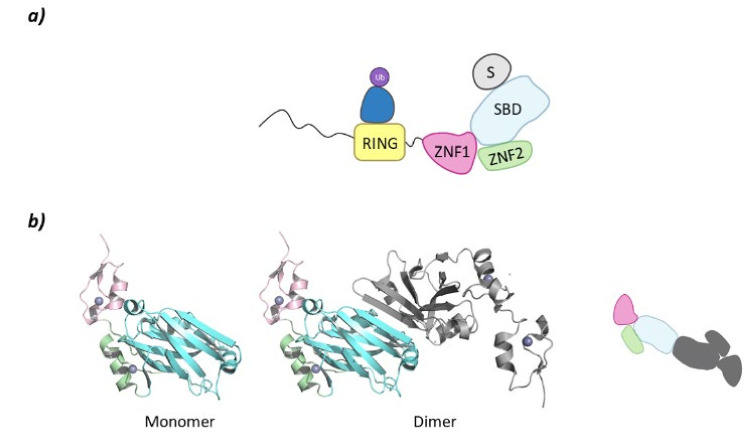
SIAH2. (**a**) Schematic representation of the assembly of a monomer of the SIAH2 E3 ligase: ubiquitin (Ub) in purple, E2 ligase (E2) in dark blue, Ring domain (RING) in yellow, zinc finger domain 1 (ZNF1) in pink, zinc finger domain 2 (ZNF2) in green, substrate binding domain (SBD) in light blue, and substrate protein in grey. (**b**) 3D representation of the dimerization of SIAH2 by the SBD as found in PDB code 5H9M. One of the monomers of the homodimer is colored following the existing color code, and for clarity, the second monomer has been colored grey.

Despite belonging to the same family, there are structural differences between SIAH1 and 2 and SIAH3. The SIAH1 and SIAH2 are structurally similar, whereas SIAH3 does not present a RING domain. SIAH2 presents a disordered region of around 80 amino acids at the *N*-terminal end, a RING domain, and two tandem Zinc fingers (ZNF1 and ZNF2). At the *C*-terminal domain lies the Substrate Binding Domain (SBD) that, in SIAH2 can dimerize as can be seen in PDB structure 5H9M (Figure 9) [124].

### 2.4. Heterodimeric RING Ligases

#### MDM

The MDM RING family is comprised of two family members: MDM2 and MDM4 (a.k.a. MDMX) that may work separately or form homodimers (MDM2/MDM2) or heterodimers (MDM2/MDMX) [125]. These are mono-subunit ligases that interact with the POI and the E2 ligase, bringing them together to favor ubiquitination.

MDM2 and MDM4 are highly implicated in cancer, as they are known to regulate the oncosuppressor protein p53. DNA damage will favor the phosphorylation of Ser429 in MDM2 that will increase autoubiquitination and degradation of the E3 ligase, thus avoiding the degradation of the oncosuppressor protein p53 [126]. It has been observed that, in many cancers, p53 is downregulated due to the overexpression of MDM2 alone or with MDM4. Interestingly, MDM2 resents higher affinity for the p53 protein than MDM4; however, in the heterodimeric MDM2/MDM4 form, the ubiquitination of p53 is enhanced [15]. Experimental evidences have demonstrated that, while the activity of MDM4 is not crucial in the regulation of the p53 protein in late stages of development, MDM4 has been found to be critical for the regulation of the p53 protein in embryogenesis by the formation of the MDM2/MDM4 complex [125]. 

**Figure 10:**
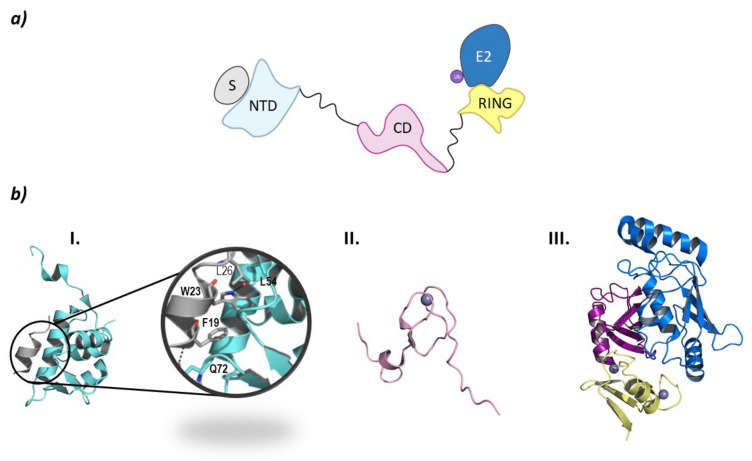

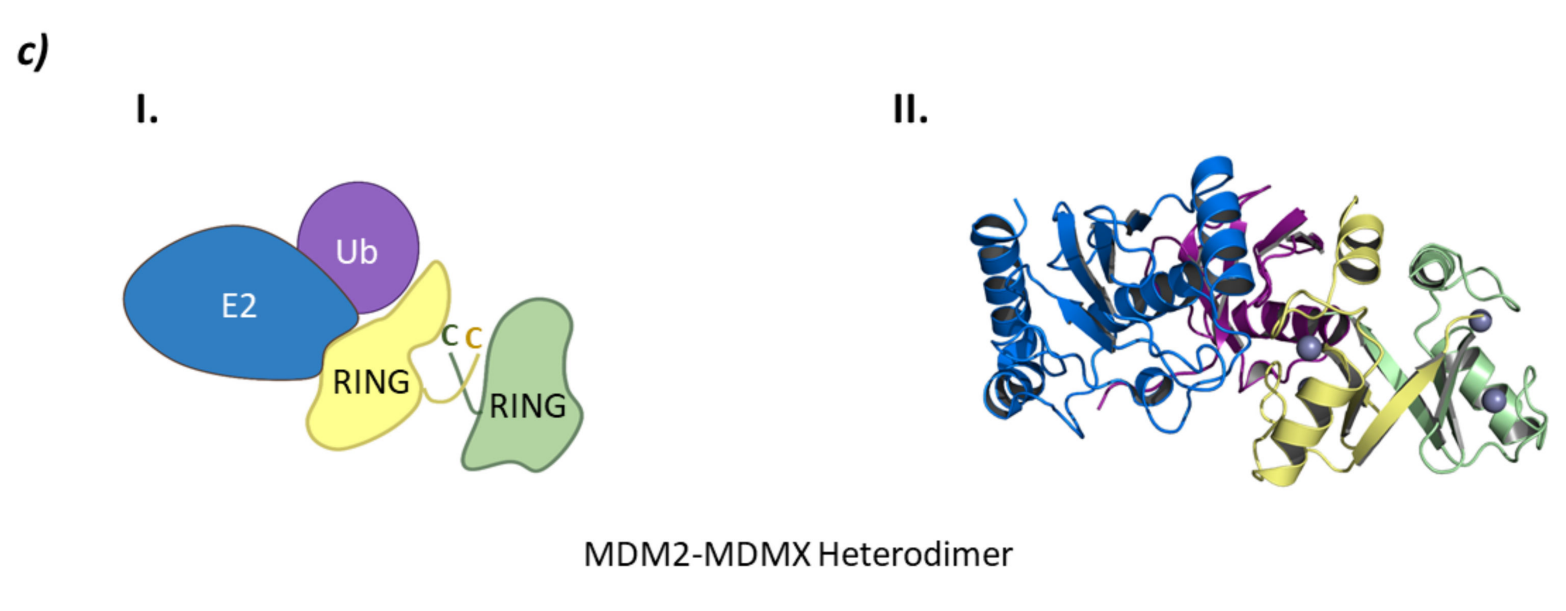
MDM. (**a**) Schematic representation of the assembly of a monomer of the MDM E3 ligase: ubiquitin (Ub) in purple, E2 ligase (E2) in dark blue, Ring domain (RING) in yellow, central domain (CD) in pink, *N*-terminal domain (NTD) in light blue, and substrate protein in grey. (**b**) Protein assembly I is the PyMOL stick and cartoon representation of the NTD of MDM in complex with a small α-helix of the p53 oncosuppressor protein as found in PDB code 4HFZ. Protein assembly II is the 3D representation of the CD as found in PDB code 2C6A. Protein assembly III is the 3D representation of the RING domain in complex with E2 ligase UbcH5B bound to ubiquitin as found in PDB code 7AI1. (**c**) I is the schematic representation of the assembly of the heterodimeric assembly of MDM2/MDMX E3 ligases: ubiquitin (Ub) in purple, E2 ligase (E2) in dark blue, Ring domain (RING) of MDM2 in yellow, RING domain (RING) of MDMX in green, central domain (CD) in pink, *N*-terminal domain (NTD) in light blue, and substrate protein in grey. Protein assembly II is the 3D representation of the heterodimeric assembly of MDM2/MDMX as found in PDB code 5MNJ.

From a structural point of view, MDM2 and MDMX are divided into three regions: an *N*-terminal end, where the binding site to p53 is located; a central domain with a zinc finger domain and an acidic domain; and a *C*-terminal end, mainly made up of the RING domain (Figure 10) [127]. The *C*-terminal domain is essential for the E3 ligase activity, as it presents the E2 binding site, as well as the site for oligomer formation. Nevertheless, for the ubiquitination and ulterior degradation of p53, both the RING domain and the acidic central domain of MDM2 become necessary [128]. Oligomer formation has been observed to take place around several hydrophobic amino acids located at the *C*-terminal ends of MDM2 and MDMX; however, the sequences differ slightly. The RING domains of the adjacent monomers interact with each other through these amino acids forming a cross-like structure (Figure 10) [129]. In this line, Metzger M. B. et al. proved that the conformation of a phenylalanine residue at the *C*-terminal end of these ligases (Phe490 in MDM2 and Phe488 in MDMX) was key for the formation of the monomeric or oligomeric form of MDM2 [130].

The p53 binding site is located at the *N*-terminal end of these ligases and is comprised of three hydrophobic pockets that interact with residues Phe19, Trp23, and Leu26 which are part of the α-helix of p53 that interacts with MDM2 and MDMX [131]. The interaction of MDM2 with p53 is stabilized by two hydrogen bonds established between F19 and W23 of p53 and Q72 and L54 in MDM2, respectively (Figure 10) [132]. The p53 binding sites of MDM2 and MDMX are very similar; nevertheless, they differ slightly in the amino acid sequence of the leucine and tryptophan pockets, which is being exploited for the design of inhibitors of the MDMX-p53 interaction [15,131]. 

Due to the implication of MDM2 in cancer regulation, there is a great interest in the development of selective inhibitors that impair the binding of the E3 ligase to the oncosuppressor protein p53 favoring cell death [133,134,135,136,137,138]. To date, inhibitors that destabilize the MDM2-p53 binding (Nutlin-3a, RG7112, MI773, amongst others) and MDMX-p53 binding (SJ-172550, WK298, amongst others) have been described, as well as a stapled peptide (ALRN-6924) that acts as a dual inhibitor of the interaction of p53 with both MDM2 and MDMX, have been described. Additionally, efforts are being made to impair the formation of the dimer of MDM2 and MDMX using small, short peptides [131]. Fan Y. et al. recollected information on these inhibitors detailing which ones have entered the clinic for the treatment of many tumors (Figure 10) [131]. MDM2 has also been found to mediate in the regulation of the androgen receptor (AR) that plays a critical role in the development of androgen dependent and independent tumors. The regulation of the AR is mediated by both the acetyltransferase-containing co-activator proteins and HDAC1. Gaughan L. et al. have demonstrated that MDM2 binds to both AR and HDAC1 and that the ubiquitination of both proteins regulates the function of the AR [139]. In addition to the role that MDM2 plays in cancer regulation, it has also been shown to ubiquitinate Lys47 of HDAC1 in vascular calcification [140]. 

## 3. Hect Ligases

The Homologous to E6AP *C*-Terminus (HECTs) E3 ligases is a smaller family of E3 ligases that are involved in a wide range of pathologies such as neurodegenerative disorders, hypertension, and cancer. This type of E3 ligases carry out a two-step ubiquitination mechanism: they first bind the Ub-E2 ligase and catalyze the transfer of the Ub onto a catalytic cysteine; secondly, they bind the substrate protein and transfer the Ub molecule. This type of mechanism voids the substrate specificity of the E2 ligases as the ubiquitination of the substrate protein will be directed by its interaction with the HECT ligase through adaptor proteins. Interestingly, HECT ligases can be hijacked by viral proteins that favor the ubiquitination of proteins that are not the natural substrates of this system [141]. The main structural characteristic of these ligases is that they present the HECT domain at the *C*-terminal end of the protein. This domain is comprised of two lobes connected by a flexible loop: the E2-binding domain at the *N*-lobe, and the *C*-lobe that contains the catalytic cysteine that binds to the Ub molecule [142]. 

The HECT family can be divided into three subfamilies depending on the characteristics of the *N*-terminal domain: the NEDD4 subfamily that presents WW and C2 domains, the HERC subfamily that presents regulator of chromatin condensation-like domains (RLD); and the “Other” subfamily that have different *N*-terminal domains that may or may not contain the WW and C2 or the RLD domains [141]. Interestingly, this family of ligases are able to build specific ubiquitin chains independently of the E2 that take part of the process [143]. 

Inhibitors of this family of ligases have been identified using phage libraries, and despite not having a clear understanding of their mechanisms of action, they all alter the Ub transfer from the E2 ligase to the catalytic cysteine of the HECT domain [143]. 

### 3.1. Smurf2

Smurf1 and Smurf2 are the two members of the Smurf (SMad Ubiquitination-Related Factor) family. The cellular location of the two Smurf family members will mainly depend on their target proteins. Interestingly, Smurf1 is mainly located in the cytoplasm, but can translocate to the nucleus, while Smurf2 acts in the opposite way; it is mainly nuclear but can translocate to the cytoplasm [144]. In normal conditions, Smurf2 is responsible for maintaining genome stability, thus acting as a tumor suppressor by regulating, among other substrates, the TFG-β pathway (Transforming Growth Factor beta) that is implicated in tumor suppression and embryonic development processes [145]. This regulation is carried out indirectly by the interaction of Smurf2 with Smad7, a member of the Smad family that are signal transducers for receptors of the TFG-β superfamily [146,147]. Interestingly, the activation of the PTH (Parathiroid Hormone) pathway in osteoblasts can trigger the Smurf2-mediated proteasomal degradation of HDAC4. The reduction in this HDAC’s levels favors the differentiation of the osteoblasts into osteoclasts by the induced expression of RANKL (Receptor Activator of Nuclear factor κ-B Ligand) brought about by the transcription factor MEF2C (Myocyte Enhancer Factor 2C) [148]. 

However, Smurf2 can either act as tumor promoter or suppressor, depending on various factors such as tumor stage and type, amongst others [149]. Smurf2 exerts its pro-oncogenic effect regulating various cell signaling pathways such as RAS, Wnt, and EGFR. Additionally, as a multiple checkpoint protein regulator (i.e NEDD9-Aurora A centrosome complex in G2 and prophase; and the Mad2 spindle checkpoint complex in prometaphase, RhoA in cytokinesis in anaphase), the lack or depletion of Smurf2 is related to the delay in mitosis [147,149]. 

The tumor suppressor activity of Smurf2 is related to the ability of Smurf2 to induce cell senescence when telomers are shortened, thus avoiding the accumulation of mutations by degrading Id1 (Inhibitor of DNA binding 1), which leads to the expression of p16 that is the ultimate senescence inducer. Additionally, Smurf2 degrades: RNF20 (RiNg finger protein 20) that is responsible for histoneH2B ubiquitination; TopoIIα (Topiisomerase IIα) that is involved in chromosomal translocations; SATB1 (AT-rich Sequence-Binding protein 1) that promotes the growth and metastasis of colon cancer; KLF5 (Transcription factor-Krüppel-like factor 5) that promotes colon and bladder cell proliferation; and YY1 (Yin Yang 1) that is a Krüppel-like zinc finger transcriptional factor expressed in various cancers [149]. 

From a structural point of view, the Smurf subfamily belongs to the NEDD4 subfamily of HECT E3 ligases, as both Smurf1 and Smurf2 present the C2 domain at the *N*-terminal end, several WW domains, and the HECT domain at the *C*-terminal end [149]. 

**Figure 11:**
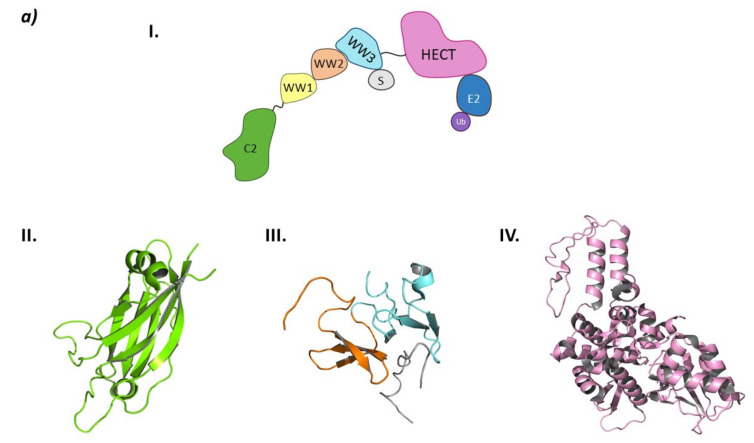

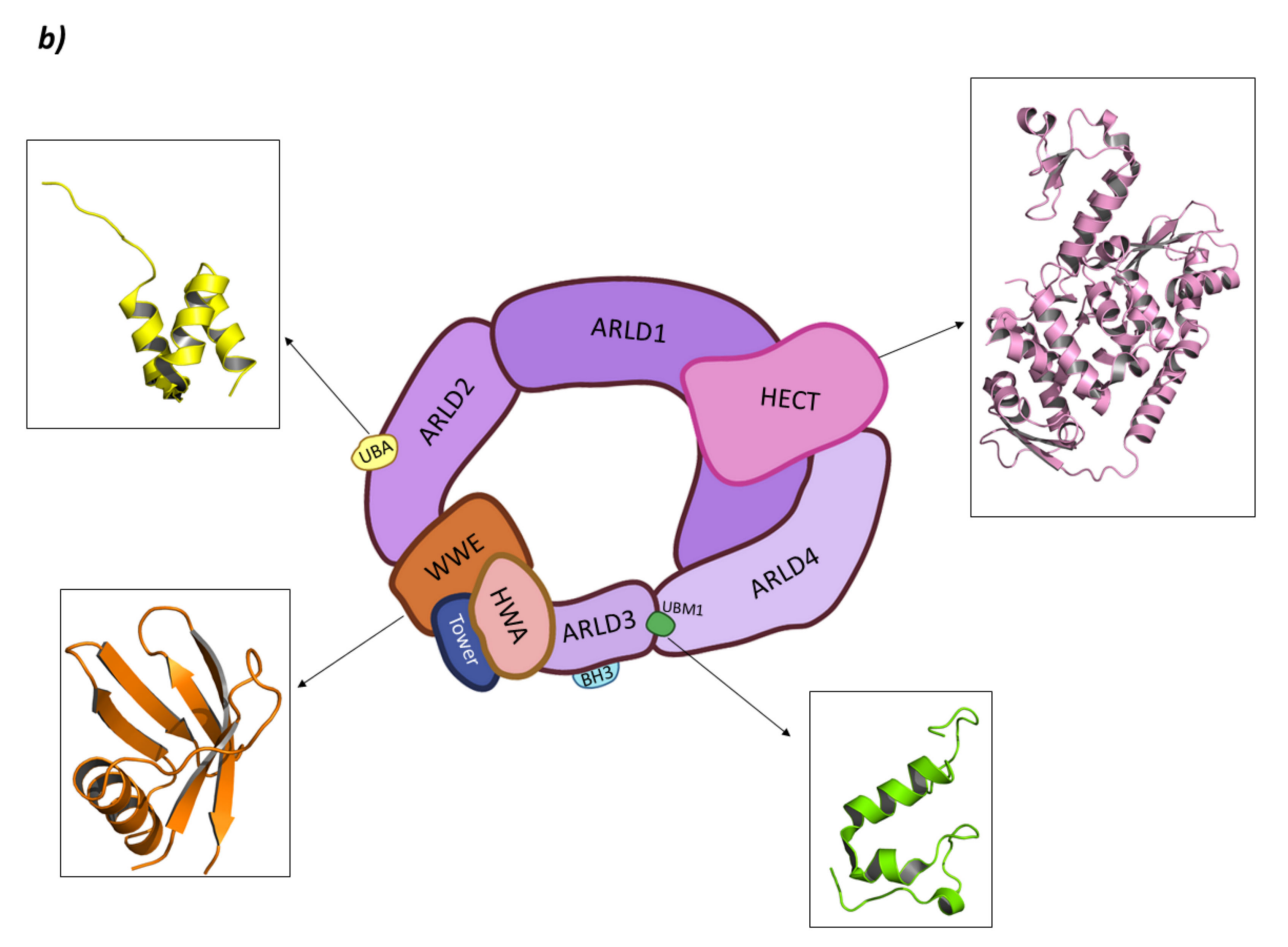
HECT E3 ligases. (**a**) Smurf2. I is the schematic representation of the assembly of the Smurf2 E3 ligase: ubiquitin (Ub) in purple, E2 ligase (E2) in dark blue, HECT domain (HECT) in pink, WW3 domain in light blue, WW2 domain in orange, WW1 in yellow, C2 domain in green, and substrate protein in grey. Protein assembly II is the 3D representation of the C2 domain as found in PDB code 2JQZ. Protein assembly III is the 3D representation of the WW2 and WW3 domains in complex with a fragment of the substrate protein Smad7 as found in PDB code 2KXQ. Protein assembly IV is the 3D representation of the HECT domain as found in PDB code 1ZVD. (**b**) HUWE1. Schematic representation of the assembly of the Smurf2 E3 ligase adapted from Hunkeler M. et al. [162]: HECT domain (HECT) in pink, armadillo repeated as domain 1–4 (ARLD1–4) in shades of lilac, ubiquitin associated domain (UBA) in yellow, tryptophan and glutamic acid-rich domain (WWE) in orange, HUWE1 WWE module associated domain (HWA) in salmon pink, tower domain in dark blue, Bcl-2 homology region 3 (BH3) in light blue, and ubiquitin binding motif 1 (UBM1) in green. The 3D representation of the individually crystalized fragments of this assembly are represented next to their position in the scheme (UBA (PDB code 2EKK) yellow, HECT (PDB code 3H1D) pink, WWE (PDB code 6MIW) orange, and UBM1 (PDB code 2MUL) green).

Structurally, Smurf2 presents the C2 domain at the *N*-terminal end, three WW domains (WW1, WW2, and WW3), and the HECT domain at the *C*-terminal end. The C2 domain presents two main activities: on the one hand, it is responsible for the auto-inactivation of Smurf2 by interacting with the HECT domain [150,151]; on the other, it allows the interaction of Smurf2 with the intracellular membranes [150]. The WW domains are responsible for target recognition, and of the three WW domains of Smurf2, WW3 is known to interact with Smad7 [146]. The interaction of the WW3 domain with Smad7 disrupts the autoinhibition of the HECT domain by the C2 domain and favors the interaction with the E2 ligase (Figure 11) [146,150]. 

### 3.2. HUWE1 (Mule)

HUWE1 is a two-faced ligase with respect to cancer, as depending on the type of cancer it will act either as an enhancer or a tumor suppressor [143,152]. Work carried out by Yang D. et al. assessed that the elimination of HUWE1 in human lung cancer cell line was linked to tumor suppression in a similar manner as the overexpression of p53 [152]. HUWE1 is also implicated in the inflammation signaling upon bacterial infection by ubiquitinating several cytoplasmic proteins in the caspase-1 pathway [153], and in neural progenitor proliferation and differentiation, cell migration, and axon development, and regulating inhibitory neurotransmission by regulating GABA receptors by ubiquitination [154]. As a two-faced ligase, HUWE1 has a controversial role in the regulation of the different oncoproteins of the MYC family, as it is able to form both Lys63 as Lys48 poliubiquitin chains. The formation of K63-poliubiquitin chains has been related to the increase in the transcriptional activity of the HUWE1 substrate proteins, whereas the K48-poliubiquitin chains induce proteasomal degradation of HUWE1 substrates. For example, *N*-Myc is Lys48-poliubiquitinated by HUWE1 in neuroblastome cells and in embryonic stem cells, thus allowing neuronal differentiation and cell cycle arrest [155,156]. Myant K. B. et al. demonstrated that in colon cancer, HUWE1 mediated in the proteasomal degradation of *c*-Myc, as the absence or mutation of this E3 ligase results in an observable increase in cell proliferation as a result of increased *c*-Myc levels [157]. On the other hand, Peter S. et al. indicated that HUWE1 formed Lys63-poliubiquitin chains in *c*-Myc, which led to the activation of its transcriptional activity and, in parallel, it also formed Lys48-poliubiquitin chains in MZ1 to induce its degradation. MZ1 is a component of the MYC/Max/MZ1 repressor complex [158]. In addition, HUWE1 is implicated in the formation of K63-poliubiquitin chains in *c*-Myc in multiple myeloma, where it can be found overexpressed [159]. Another HUWE1 substrate is Mcl-1, whose proteasomal degradation allows apoptosis as a response to DNA damage [160]. Moreover, HUWE1 is in charge of the ubiquitination of tumor-involved histone H1.3, or physiologically involved histones H1 and H3, amongst others. Although it may seem contradictory, it can increase the deubiquitinase activity of enzyme USP7 by the formation of Lys63-poliubiquitin chains [156].

HUWEI has also been found to be implicated in an intricated p53-regulating pathway that involved different types of E3 ligases, tumoral suppressors and HDAC2. In fact, p53 and HDAC2 are two targets of this ligase [116,153]. p14ARF is a tumor suppressor that is responsible for the activation of p53 functions in a HUWE1 and MDM2-dependent manner by the inhibition of both E3 ligase activities [156].

HUWE1 belongs to the “Other” subfamily of HECT E3 ligases whose *N*-terminal end is still being studied. In 2018, Jasper Sliumer and Ben Distel described the *N*-terminal end to have four domains: ARM (ARMadillo-type fold domain) which is believed to mediate in vesicular transport of many proteins; the UBA domain (UBiquitin Associated domain) which is responsible for binding polyubiquitin chains; the WWE domain (Triptophan and Glutamic acid-rich domain) which is responsible for protein–protein interactions during the ubiquitination phase; and the BH3 domain (Bcl-2 Homology region 3) which specifically recognizes the Bcl2-family member Mcl-1 [141,154,160,161]. However, in 2020, Andrew C. Giles and Brock Grill published a review article where they refuted the existence of the ARM domain, postulating that instead two Domains of Unknown Function (DUF908 and DUF913) occupied that region of the protein. Additionally, in the *C*-terminal end of the BH3 domain, they indicated the presence of the UBM, the function of which is yet to be fully elucidated [154]. Nevertheless, almost at the same time, Hunkeler M. et al. obtained the cryo-EM structure of HUWE1 where they described the *N*-terminal end. According to this study, this end is made up of four Armadillo Repeated Liked Domains (ARLD1–4); a WWE domain; and a “Tower” and HWA (HUWE1 WWE module Associated) domain [162]. The ARLD2 contains UBA (UBiquitin Associated) domain and UIM (Ubiquitin Interacting Motif) being the first involved in the binding of polyubiquitin chains, whereas the activity of the second is still not clear [162]. Moreover, BH3 domain is inserted in the ARLD3 domain. Additionally, they demonstrated that the active form of HUWE1 presents a ring-like shape that closes through the interaction of the ARLD1 and ARLD4 domains that interact face to face while the HECT domain is located on top of them. In fact, mutations of these domains lead to a loss of the HUWE1 activity, putting forward the relevance of the closed ring-like structure (Figure 11) [162]. 

Interestingly, when other HECT family members present an inhibited conformation in which one of the domains in the *N*-terminal end interacts with the HECT domain, HUWE1 can either do so in a similar way or by oligomerization. The structural basis of the inhibition of HUWE1 is quite convoluted. The *C*-terminal domain has been found crystalized, forming an asymmetric dimer, but a small amino acid sequence before the *C*-terminal domain is responsible for the inhibition impairment of HUWE1. Additionally, tumor suppressor p14ARF works as a HUWE1 inhibitor by interacting with that small amino acid segment and hijacking the enzyme in the auto-inhibited dimer conformation [141,154,163].

## 4. Known PROTACs for HDACs

**Figure 12:**
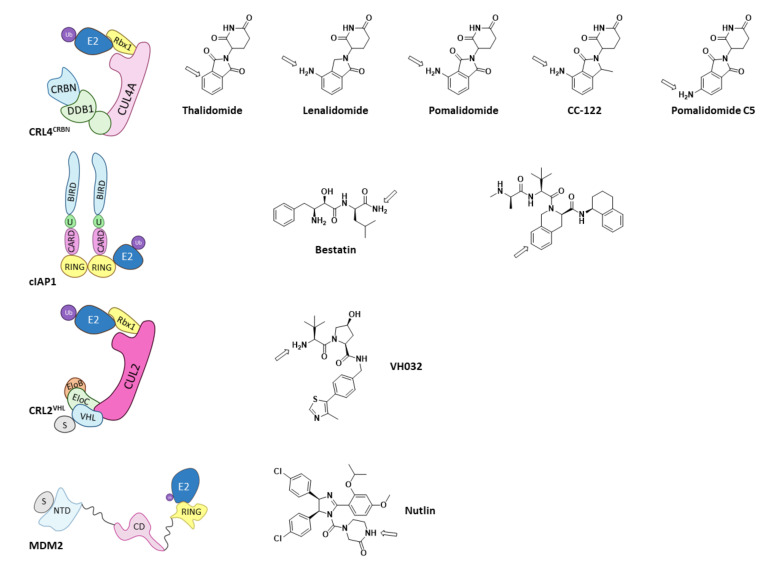
Schematic representation of the assembly of the E3 ligases that have been used for PROTAC design and the 2D representation of the structures of their respective ligands with and arrow marking the position for the functionalization. Adapted from Scheepstra M. et al. [38].

**Figure 13:**
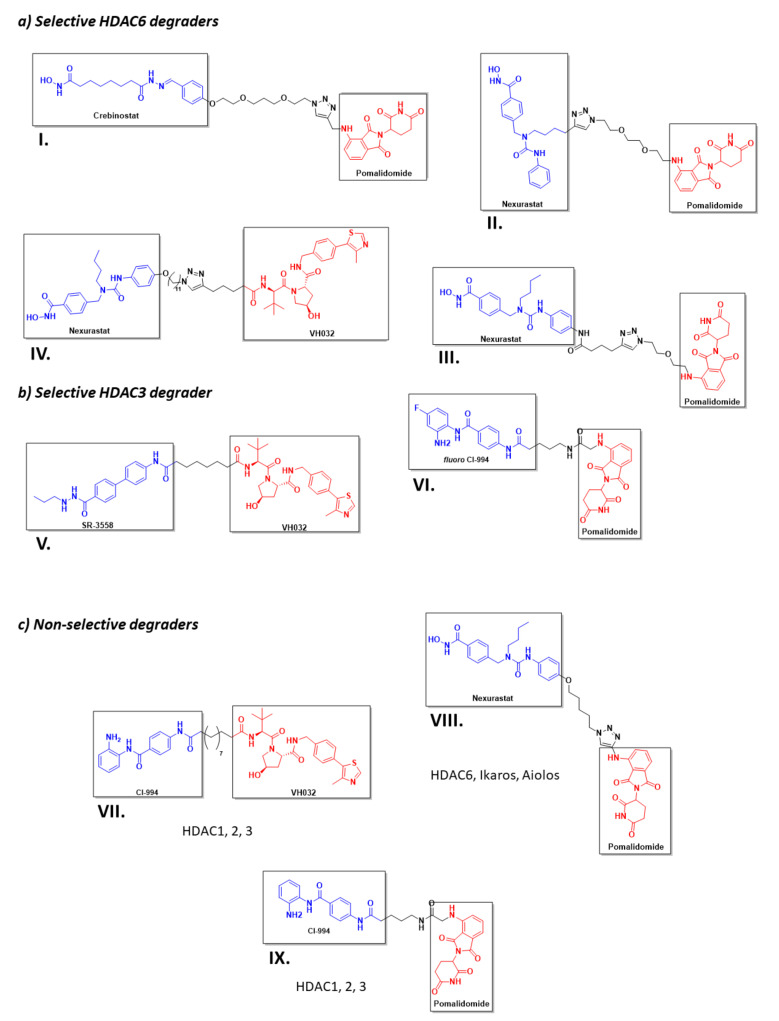
2D representation of the structures of the known HDAC-directed PROTACs [60,61,166,167,168,169,170,172] adapted from Smalley J. P. et al. [75].

Of all the existing E3 ligases, only a few have proven to be valid for PROTAC design. As depicted by Scheepstra M. et al. in their 2019 review, the PROTAC-targeted ligases are CRL4^CRBN^, cIAP1, MDM2, and CRL2^VHL^, as they present known ligands that can be functionalized to add the needed linker and POI ligand (Figure 12) [38]. Arrows in Figure 13 indicate the most common position for the functionalization of these inhibitors; however, other positions can be succesfully functionalized for the obtention of different PROTACs, as very recently hightlighted by Bricelj A. et al. [164]. Of all the E3 ligases that can be hijacked by PROTACs, in order to achieve selective degradation of HDACs, only CRL4^CRBN^ and CRL2^VHL^ have been targeted [38,60,61,75,165,166,167,168,169,170]. Interestingly, neither of these two ligases naturally target HDACs, but have proven to be prone to hijacking for the degradation of non-natural substrates [38]. 

Regarding HDAC inhibitors, they all present a Zinc-Binding Group (ZBG) a linker and a cap group (CG). The ZBG targets the catalytic zinc ion, while the linker establishes interactions with the amino acids lining the sides of the access tunnel to that ion; and, the cap group interacts with the surface of the protein [171]. Despite the difficulties for achieving selectivity derived by the homology between HDAC family members, different structural modifications to the basic ZBG-linker-CG have proven to be useful for selective targeting of the different HDACs, as has been gathered by Melesina J. et al. in their review on HDAC inhibitors [171]. 

However, despite the amount of described HDACis, only a few have been used as POI ligands in the design of HDAC targeted PROTACs: Nexturastat A^®^ (potent and selective HDAC6 inhibitor [171]), Crebinostat^®^ (pan-HDACi) [75], CI-994, and SR-3558 (Class I HDAC inhibitors) [165]. The design of PROTAC I (Figure 13) using the pan-HDAC inhibitor crebinostat and pomalidomide resulted in a selective reduction in the cellular levels of HDAC6 at 80 nM concentrations in MM.1S cells [38,75,168], and in a dose-dependent manner, where the optimal effect was found at concentrations ranging from 123 nM to 370 nM in MCF-7 cells [38,168]. Additionally, PROTACs II and III (Figure 13) designed by Smaley J.P. et al. produced an HDAC6 knockdown in different cell lines at 100 nM [75]. More precisely, PROTAC II produced a higher effect in the MM.1S cell line with a lethal dose of 3.8 nmol/L by specific degradation of HDAC6 [166]. A similar Nexurastat A^®^-based PROTAC (Figure 13, PROTAC VIII) by Zixuan A. et al. resulted not only in HDAC6 depletion at 30 nM, but was also able to deplete Ikaros and Aiolos, two known CRL4^CRBN^ natural substrates [75,167]. As a result, PROTAC IV was designed with a longer linker to target the CRL2^VHL^ E3 ligase to avoid off target degradations of CRL4^CRBN^ substrates, while maintaining the ability to degrade HDAC6 at a DC_50_ (Degradation Concentration) of 7.1 nM in MM.1S cells [75].

Selective, long-lasting, but reversible HDAC3 dose-dependent degradation was achieved by PROTAC V (Figure 13) in MDA-MB-468 cells with a DC_50_ of 42 nM [61,75]. Interestingly, Cao et al. designed a CI-994-based PROTAC (Figure 13, PROTAC VI) that, by modulating the length and nature of the linker, achieved HDAC3 selectivity over HDAC 1 and 2. A fluorine atom next to the ZBG played a crucial role in the selectivity [172]. PROTAC VII was able to degrade around 50 % of HDACs 1 to 3 in HCT116 colon cancer cells at 1 μM concentrations [75]. 

It is noteworthy to highlight that, in the attempt to design a cIAP1-based HDAC directed PROTAC using SAHA as HDAC binding moiety, and bestatin as E3-ligase recruiter, instead of achieving PROTAC degradation, the resulting compounds proved to be strong dual inhibitors. Bestatin is also an approved AminoPeptidase N (APN) inhibitor, and the resulting hybrid molecules showed a greater inhibitory activity against APN than bestatin. Moreover, the inhibitory activity against HDACs 1, 6, and 8 of the hybrids was better than that of SAHA. Decreased HDAC protein levels were found in cellular assays, but this depletion was not due to the PROTAC effect of the hybrid molecules, and was similar to that found when using SAHA [173].

## 5. Conclusions and Future Work

Selective HDAC inhibition has become a popular topic in drug discovery, as HDACs are implicated in the development of many types of cancer, diabetes, and inflammatory, neurodegenerative, and cardiovascular diseases. In recent years, many researchers have attempted to address this challenge by the design and synthesis of HDAC-directed PROTACs. The PROTAC strategy requires less selectivity and/or potency to produce HDAC degradation than classical inhibitors. PROTAC I (Figure 13) can induce HDAC6 selective degradation despite having a non-selective inhibitor in its structure. As a result of the catalytic mechanism of action of the PROTACs, lower doses are needed to produce the same effect, thus reducing the possibilities of adverse effects when formulated as medicines [28]. As mentioned above, PROTACs are a very promising approach, although they have some limitations. It is important to optimise the length and nature of the linker in order to form a stable ternary complex (HDAC:PROTAC:E3) for selective HDAC degradation. However, this feature will vary depending on the E3 ligase. If the hijacked E3 ligase is CRL2^VHL^, the length of the PROTAC linker should be longer than if the ligase is CRL4^CRBN^ [60]. In addition, there are very few ligands described to bind to the different E3 ligases. This factseverely limits the design of this type of molecules [174]. In fact, only two PROTACs have reached clinical trials for the treatment of breast and prostate cancer, but none are directed for the selective degradation of HDACs [175].

This review has attempted to shed light onto the structure of the different E3 ligases that naturally control the HDAC protein levels in the cell, in order to pave the way for the design and synthesis of new HDAC-directed PROTACs.

## 6. Materials and Methods

The bibliographic search was carried out using the Google and Google Scholar search engines combined with different databases such as PubMed (https://pubmed.ncbi.nlm.nih.gov, last accessed on 8 September 2021, Web of Science (www.webofscience.com, accessed on 30 July 2021), or Scopus (www.scopus.com, accessed on 30 July 2021). The structural information of the different E3 ligases was obtained from the Protein Data Bank (www.rcsb.org, accessed on 30 July 2021) and UniProt (www.uniprot.org, accessed on 30 July 2021).

The schematic representations were created using BioRender (www.biorender.com, accessed on 3 September 2021). The 3D images of the proteins were created using PyMOL (The PyMOL Molecular Graphics System, Version 2.0 Schrödinger, LLC), and all of the chemical structures were drawn using ChemDraw 19 (www.perkinelmer.com).

The following strategies and search terms were used to address the topic: HDAC-PROTAC, proteasomal degradation of HDACs, and E3 ligases ubiquitinating HDAC (RLIM, MDM2, HUWE1, and CRL4CRBN, amongst others).

## Figures and Tables

**Table 1:** List of the E3 ligases implicated in the ubiquitination of the different HDAC isoforms.

E3 Family	E3 Subfamily	Implicated Ligase	HDAC	PROTAC
RING	Cullin RING	Cul3-REN^KCTD11^	1	
		CRL2^VHL^	1 ^†^, 2 ^†^, 3 ^†^, and 6 ^†^	YES
		CRL4^CRBN^	1 ^†^, 2 ^†^, 3 ^†^, and 6 ^†^	YES
		Cul3^SPOP^	6	
	Monomeric RING	CHFR *	1 and 2	
		PIRH2 (RCHY1) *	1 and 2	
		RLIM (RNF12) *	2	
	Homodimeric RING	SIAH2	3	
	Heterodimeric RING	MDM	2	
HECT	NEDD4	Smurf2	4	
	Others	HUWE1 (Mule) *	2	

* Ligases that have not yet been fully classified. ^†^ HDACs degraded by CRL2VHL and CRL4CRBN are not natural substrates of these ligases, and their degradation is PROTAC-mediated [38].

## Data Availability

No Data.
